# Supplementary figures and images for: Learning-related congruent and incongruent changes of excitation and inhibition in distinct cortical areas
Source: PLoS Biol. 2022 May 31;20(5):e3001667. doi: 10.1371/journal.pbio.3001667 (PMC9187120; doi:10.1371/journal.pbio.3001667)

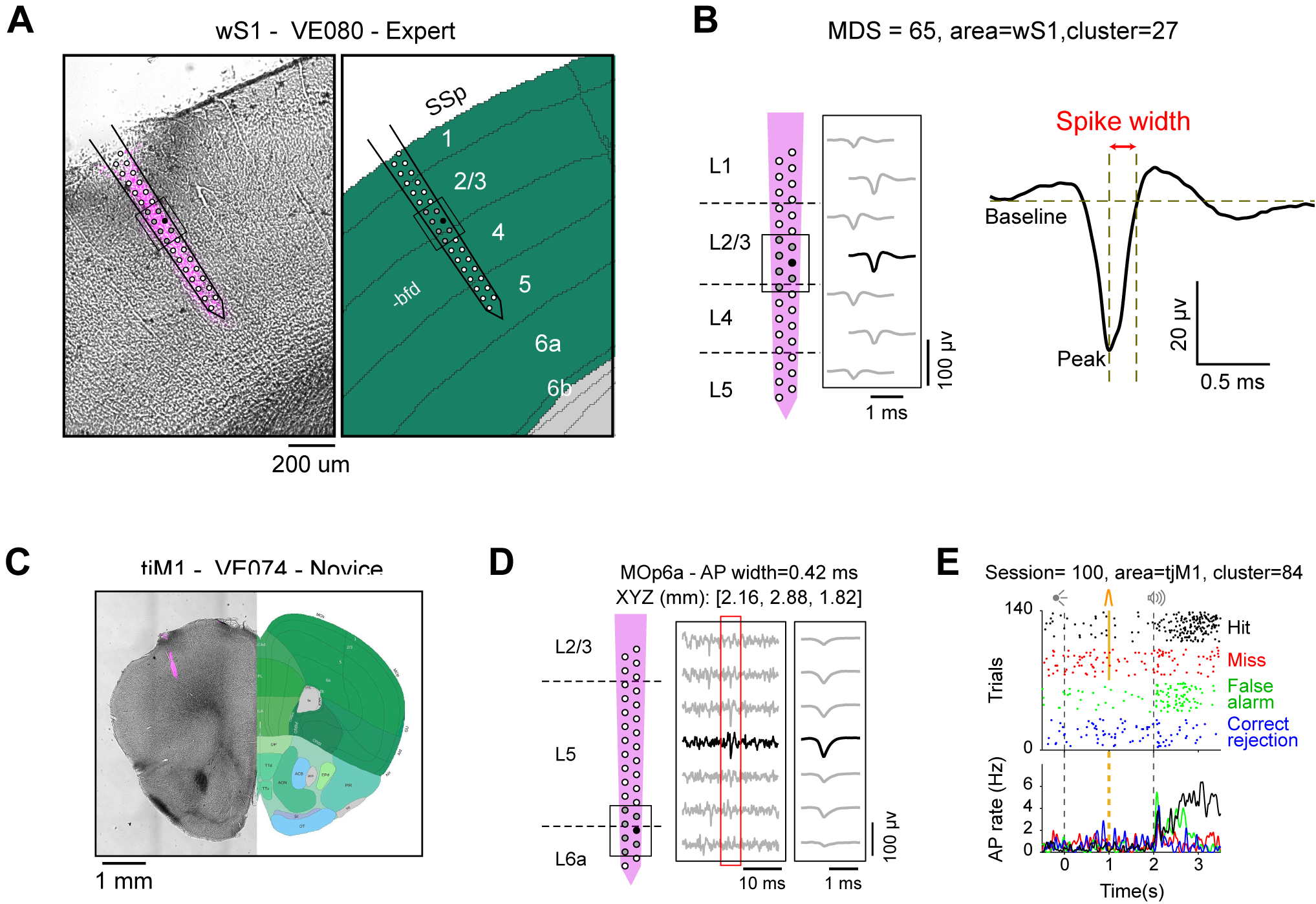

Supplement: S1 Fig — (A) Magnified example fluorescent track of the silicon probe in wS1 shown in Fig 1D and location of different probe sites after registration to the Allen Mouse Brain Atlas, https://mouse.brain-map.org. The small rectangular box and black recording site highlight the location of the example neuron shown in (B). (B) Silicon probe, example shown in (A), with site locations across cortical layers and example neuron recorded on the probe. Spikes from each neuron were observed across several sites (shown with circles) of the silicon probe. For calculating the spike width for each neuron, the average spike waveform extracted from the recording site with the largest spike peak amplitude (filled circle) was used. Spike width was defined as the time between the spike peak (minimum) to the time voltage came back to baseline level. Gray horizontal line shows spike baseline, and vertical lines mark where spike width was measured. (C) Example coronal section of a Novice mouse brain with fluorescent track of a single shank silicon probe in tjM1, registered to the Allen Mouse Brain Atlas, https://mouse.brain-map.org. (D) Reconstructed location of different recording sites of the example silicon probe shown in (C) according to Allen Atlas (left), filtered recorded raw data of 7 probe sites around one detected spike, and average extracted spike waveform for this example neuron (right). After spike sorting, the position of each neuron was assigned to the location of recording site across the probe with the largest spike amplitude (filled circle). (E) Raster plot and PSTH for the example neuron shown in (D). Trials are grouped and colored based on trial outcome. The underlying data for S1 Fig can be found in S1 Data. PSTH, peri-stimulus time histogram; tjM1, tongue-jaw primary motor cortex; wS1, whisker primary somatosensory cortex. (TIF) [file pbio.3001667.s001.tif]

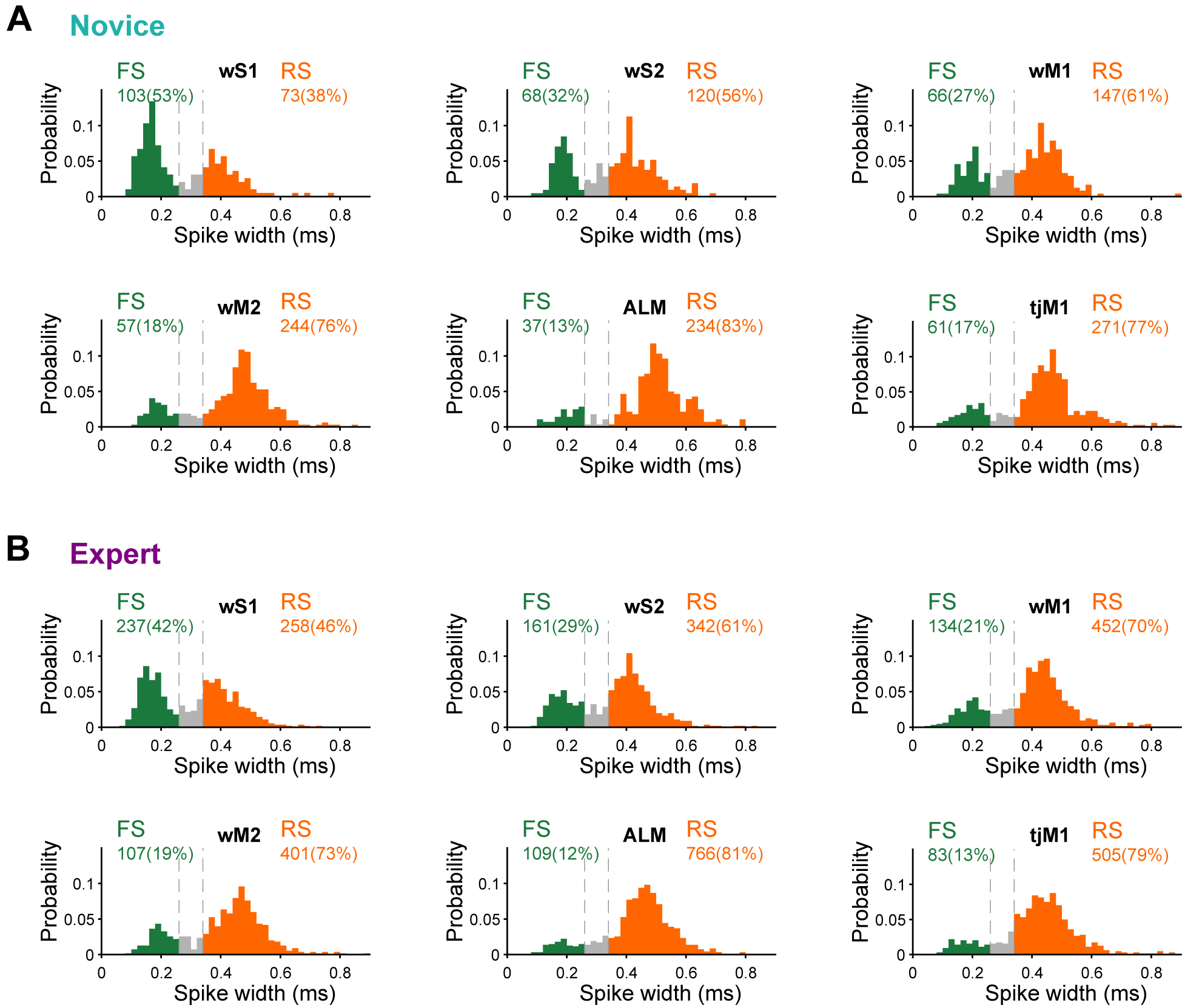

Supplement: S2 Fig — (A) Spike width distribution for neurons recorded from Novice mice shown separately for different cortical regions. Neurons were categorized as FS (spike width < 0.26 ms) or RS (spike width > 0.34 ms) in all areas. Neurons with intermediate spike width (gray bars) were excluded from the rest of analysis. Percentage of neurons in each area tagged as RS or FS are shown. (B) Same as (A) but for Expert mice. A smaller percentage of FS neurons appears to be found in frontal regions in both Novice (A) and Expert mice (B). The underlying data for S2 Fig can be found in S1 Data. FS, fast spiking; RS, regular spiking. (TIF) [file pbio.3001667.s002.tif]

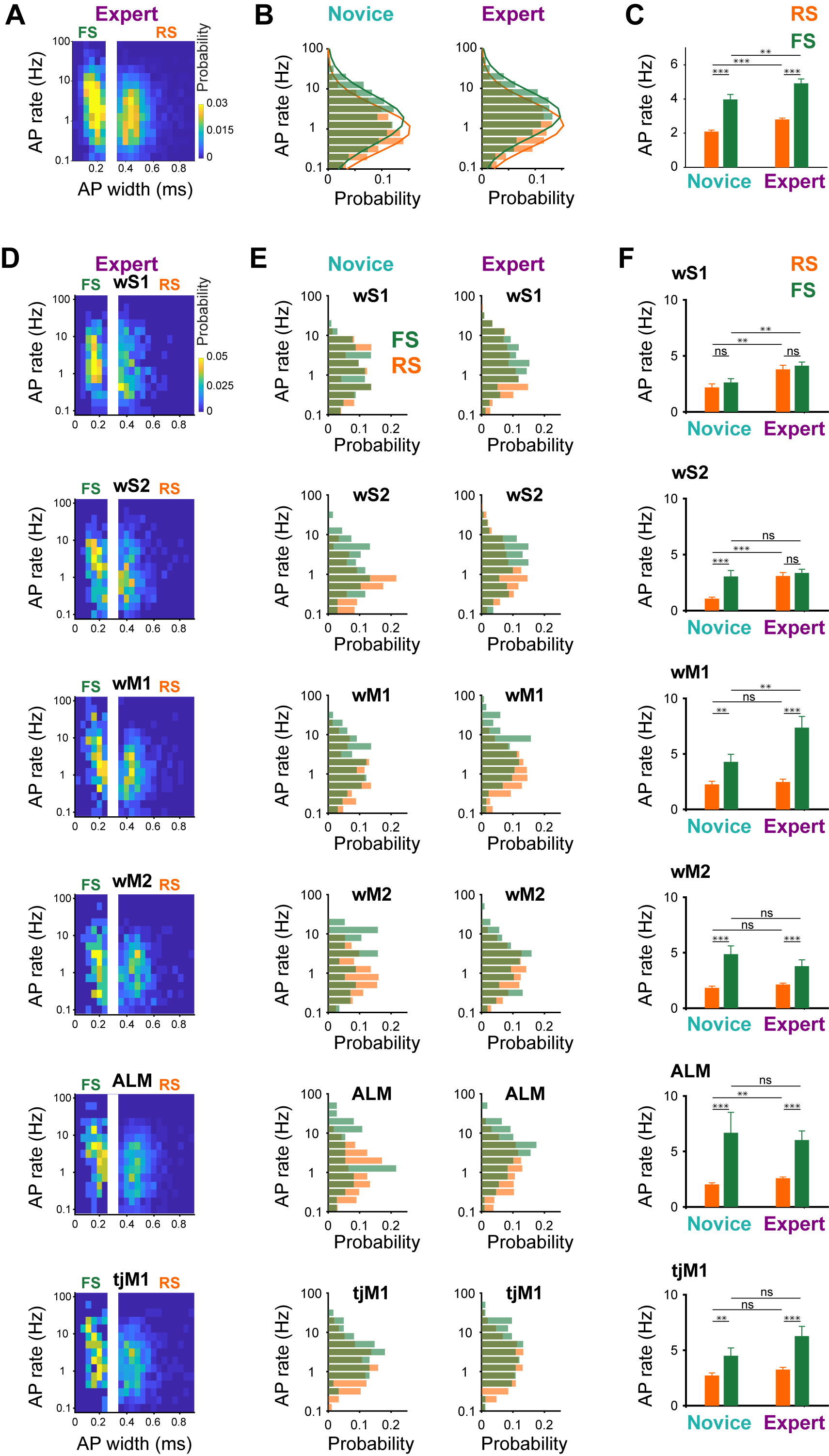

Supplement: S3 Fig — (A) Spike width distribution versus spike rate for all neurons recorded from Novice mice. (B) Spike rate distribution for RS and FS units in Novice (left) and Expert (right) mice. Note the log-normal distribution of baseline firing rates for both RS and FS units in Novice and Expert mice. Normal distributions were fit to the RS and FS histograms (solid lines). (C) Comparison of mean spike rate in RS versus FS neurons of Novice and Expert mice. Error bars: SEM. ***: p < 0.001, **: p < 0.01, *: p < 0.05, ns: p > = 0.05, nonparametric permutation test, FDR-corrected for multiple comparison. (D–F) Same as (A–C), but showing data separately for different cortical areas. The underlying data for S3 Fig can be found in S1 Data. FDR, false discovery rate; FS, fast spiking; RS, regular spiking. (TIF) [file pbio.3001667.s003.tif]

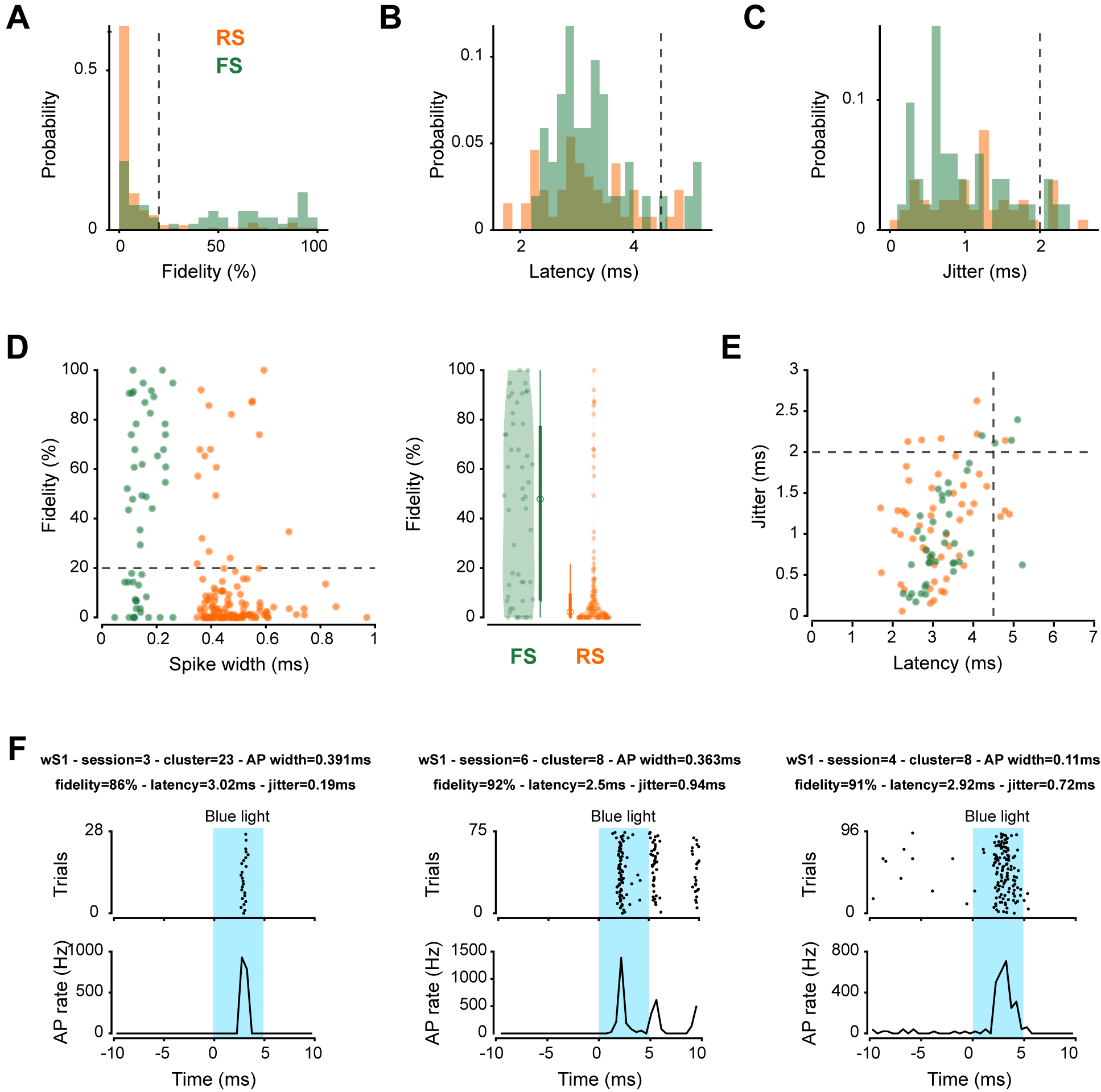

Supplement: S4 Fig — (A–C) Criteria for labeling neurons as opto-tagged upon blue light stimulation in VGAT-ChR2 mice. Probability distribution of fidelity scores (A), first spike latency (B), and jitter (C) for RS units (orange, spike width >0.34 ms, 130 neurons from 4 mice) and FS units (green, spike width < 0.26 ms, 51 neurons from 4 mice) measured in the first 10-ms window of blue light stimulation. The thresholds for detection of opto-tagged cells (dotted vertical lines) were defined based on previous literature and dips in the observed probability distributions (i.e., fidelity >20%, latency <4.5 ms, jitter <2 ms). (D) Scatter plot of spike width versus fidelity (left) and distribution of fidelity scores shown with violin plots and bar plots (right). (E) Scatter plot of latency versus jitter of light-evoked response for RS and FS units. (F) Raster plot and PSTH during the first 10 ms of 100-Hz blue light stimulation for 3 example opto-tagged neurons. The underlying data for S4 Fig can be found in S1 Data. ChR2, channelrhodopsin-2; FS, fast spiking; PSTH, peri-stimulus time histogram; RS, regular spiking; VGAT, vesicular GABA transporter. (TIF) [file pbio.3001667.s004.tif]

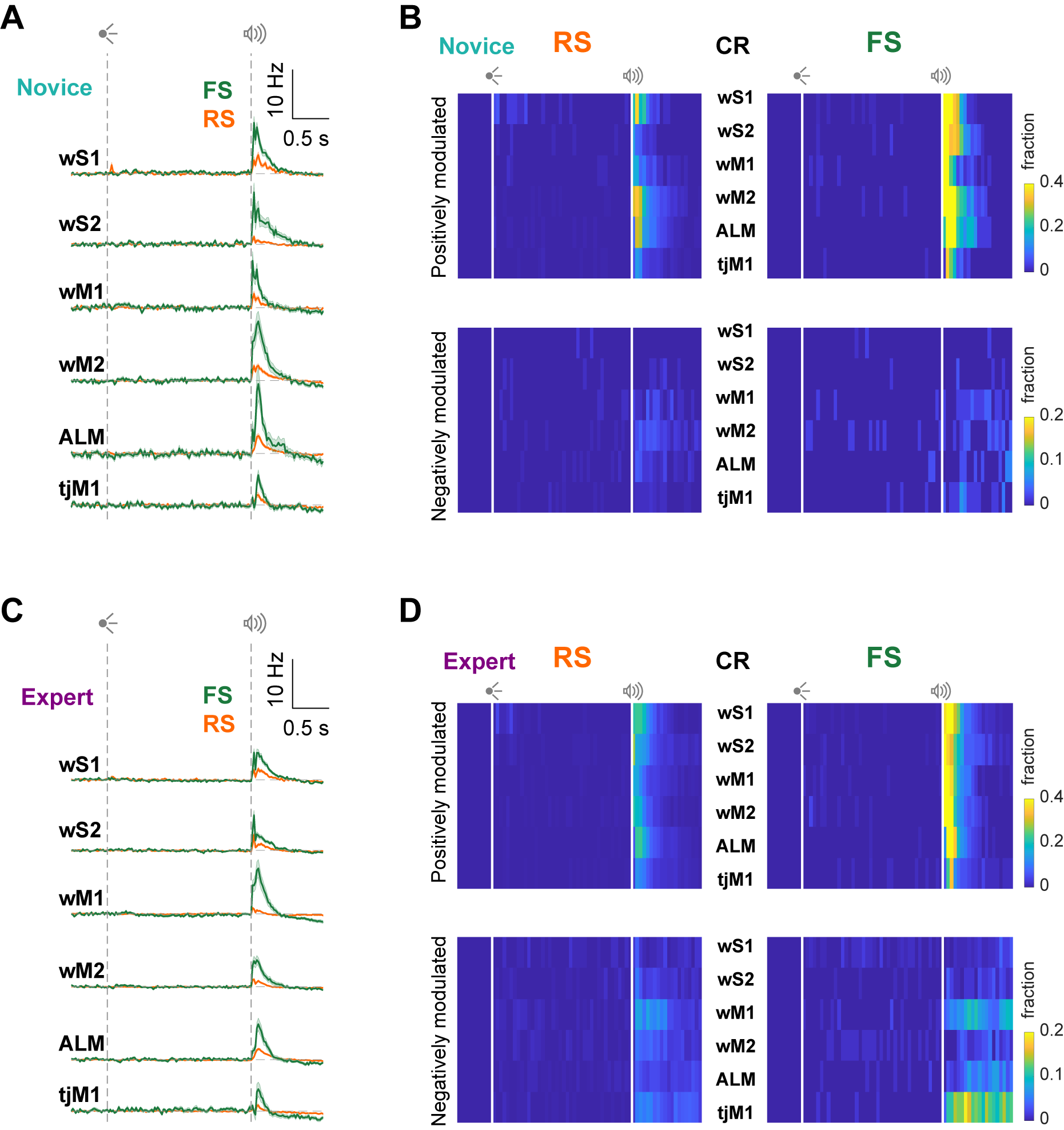

Supplement: S5 Fig — (A) Baseline-subtracted (2 seconds prior to visual onset) population firing rates (mean ± SEM) of RS and FS neurons from different regions of Novice mice are superimposed in correct rejection trials. wS1: 73 RS units in 7 mice, 103 FS units in 7 mice; wS2: 120 RS units in 8 mice, 68 FS units in 8 mice; wM1: 147 RS units in 7 mice, 66 FS units in 7 mice; wM2: 244 RS units in 7 mice, 57 FS units in 7 mice; ALM: 234 RS units in 6 mice, 37 FS units in 5 mice; tjM1: 271 RS units in 8 mice, 61 FS units in 8 mice. (B) Percentage of RS (left) and FS (right) neurons in different regions of Novice mice that are positively (top) or negatively (bottom) modulated compared to baseline (nonparametric permutation test, p < 0.05) in correct rejection trials. (C) Similar to (A), but for Expert mice. wS1: 258 RS units in 15 mice, 237 FS units in 15 mice; wS2: 342 RS units in 12 mice, 161 FS units in 12 mice; wM1: 452 RS units in 11 mice, 134 FS units in 11 mice; wM2: 401 RS units in 10 mice, 107 FS units in 10 mice; ALM: 766 RS units in 12 mice, 109 FS units in 12 mice; tjM1: 505 RS units in 11 mice, 83 FS units in 11 mice. (D) Similar to (B), but for Expert mice. There appears to be stronger suppression of both RS and FS neurons of tjM1 during the response window in Expert compared to Novice mice. The underlying data for S5 Fig can be found in S3 Data. ALM, anterior lateral motor cortex; FS, fast spiking; RS, regular spiking; tjM1, tongue-jaw primary motor cortex; wM1, whisker primary motor cortex; wM2, whisker secondary motor cortex; wS1, whisker primary somatosensory cortex; wS2, whisker secondary somatosensory cortex. (TIF) [file pbio.3001667.s005.tif]

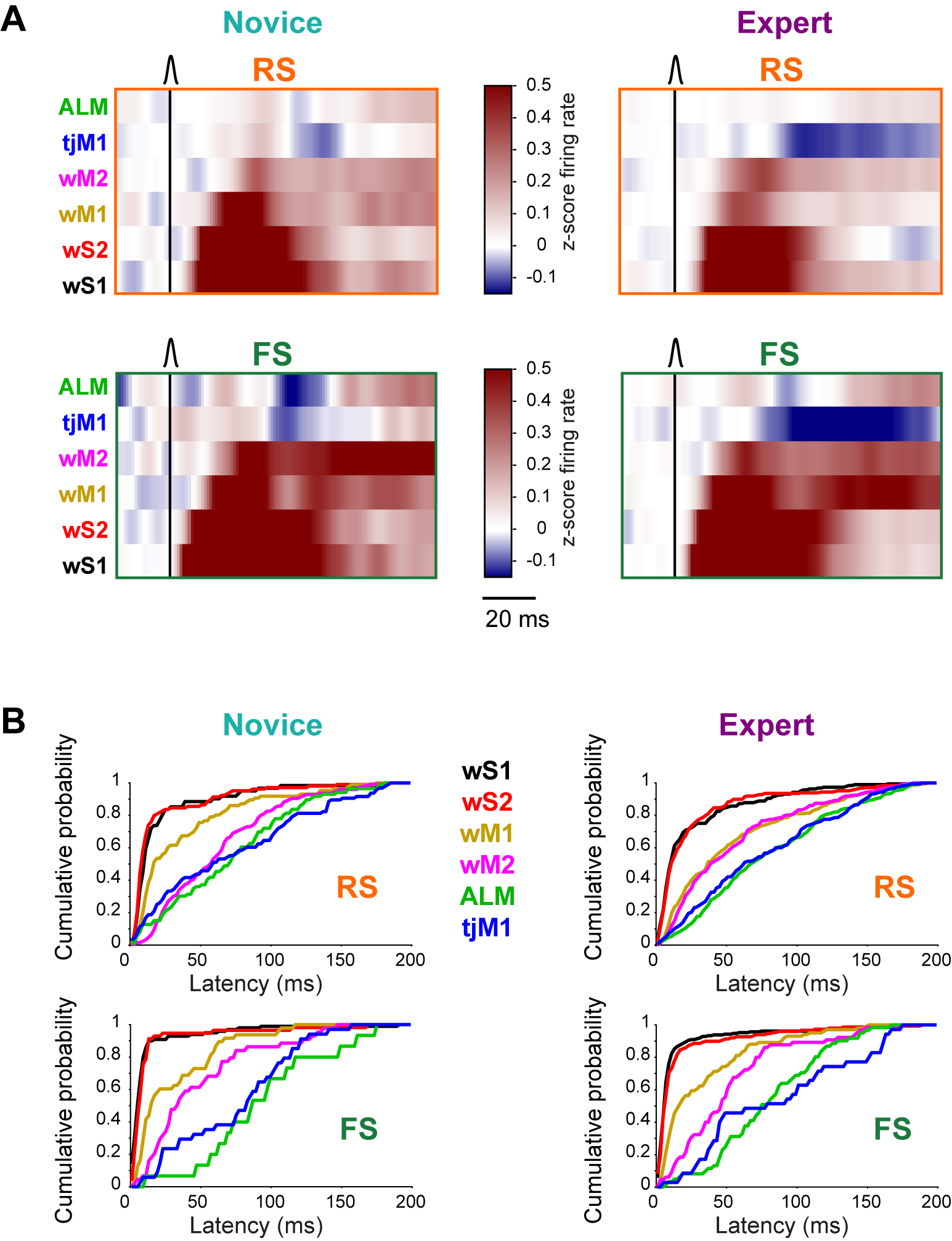

Supplement: S6 Fig — (A) Sequential propagation of whisker-evoked spiking responses in hit trials for RS (top) and FS (bottom) neurons from Novice (left) and Expert (right) mice. Mean z-scored firing rate in the first 100-ms window after whisker stimulus is shown. Brain regions are sorted based on their population-average onset latency in RS neurons of Expert mice. (B) Cumulative distribution of latency of individual neurons for different cortical areas in RS (top) and FS (bottom) neurons from Novice (left) and Expert (right) mice. Only neurons with significant modulation in the 200-ms window following whisker stimulus compared to a 200-ms window prior to the whisker stimulus are included (p < 0.05, nonparametric permutation test). Latency was defined at the half maximum (minimum for suppressed neurons) response within the 200-ms window. The underlying data for S6 Fig can be found in S4 and S5 Data. FS, fast spiking; RS, regular spiking. (TIF) [file pbio.3001667.s006.tif]

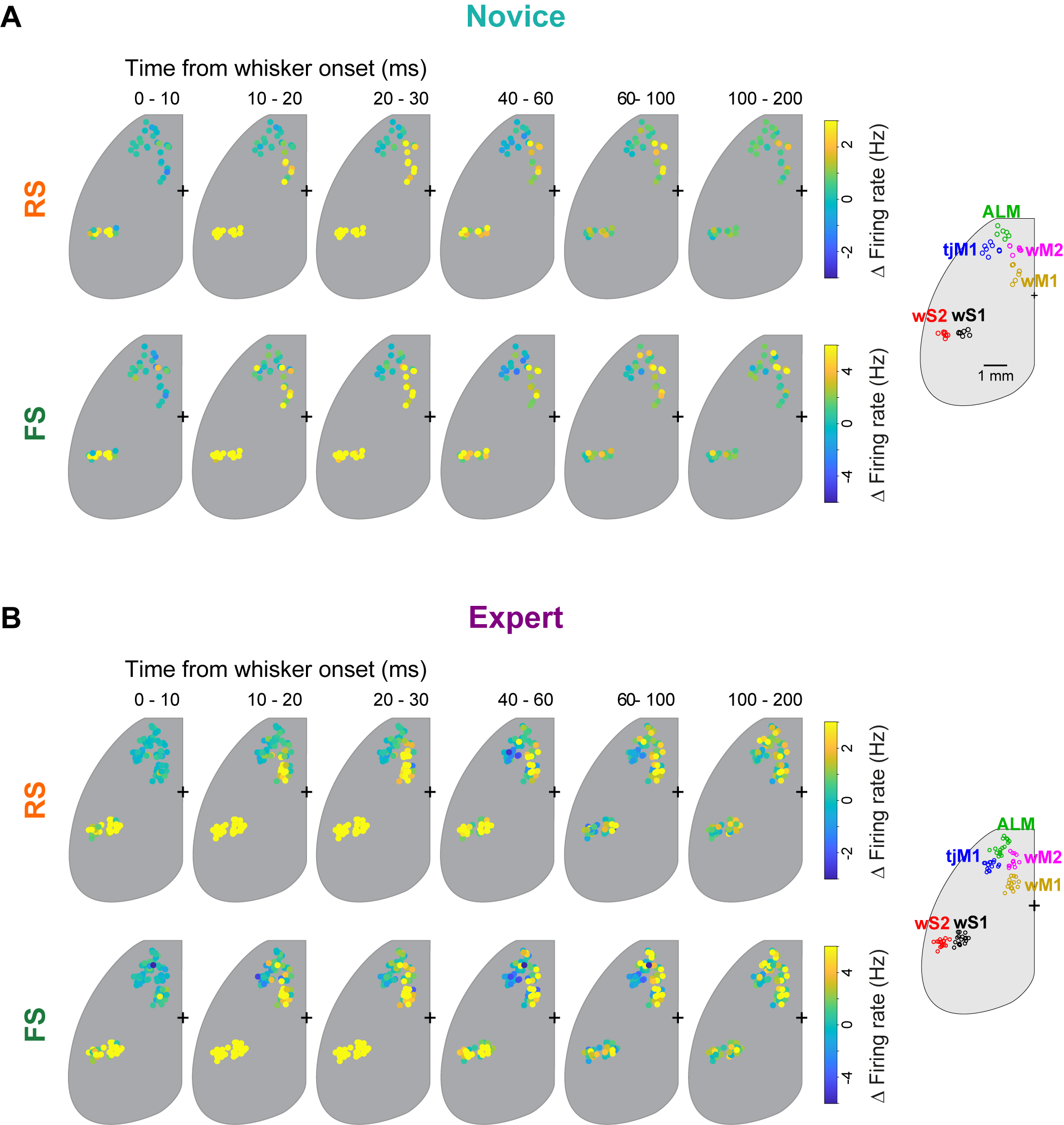

Supplement: S7 Fig — (A) Time-lapse maps of whisker-evoked firing rate in RS (top) and FS (bottom) neurons of Novice mice in hit trials. Circles represent different probes and colors show mean baseline-subtracted (50 ms before whisker onset) firing rate across each probe at different time windows. Probes from all Novice mice (43 probes in 8 mice) are superimposed. (B) Same as (A) but for Expert mice (90 probes in 18 mice). The underlying data for S7 Fig can be found in S4 and S5 Data. FS, fast spiking; RS, regular spiking. (TIF) [file pbio.3001667.s007.tif]

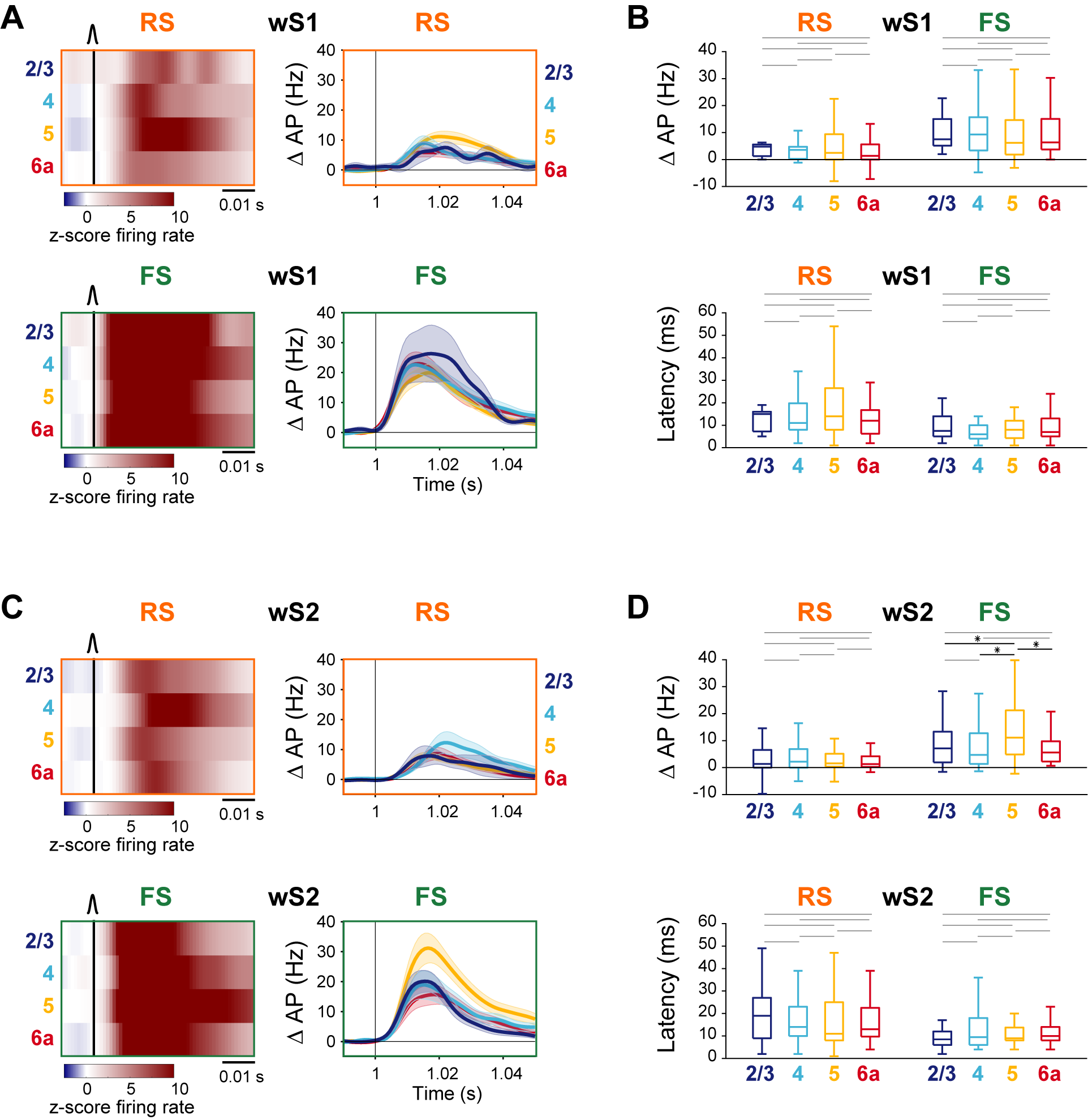

Supplement: S8 Fig — (A) Mean z-scored firing rate (left) and baseline-subtracted firing rate (right) of RS (top) and FS (bottom) neurons across different cortical layers of wS1. (B) Boxplots representing the distribution of firing rate change (top) and response latency (bottom) of RS and FS units in different layers of wS1. Only neurons with a significant whisker response in the first 100 ms (compared to 100 ms before whisker onset, nonparametric permutation test, p < 0.05) were included. Midline represents the median, bottom and top edges show the interquartile range, and whiskers extend to 1.5 times the interquartile range. *: p < 0.05. Gray lines show nonsignificant comparisons. Firing rate change was compared using nonparametric permutation test and latencies were compared using Wilcoxon rank-sum test. (C, D) Same as (A, B), but for wS2 neurons. The underlying data for S8 Fig can be found in S6 Data. FS, fast spiking; RS, regular spiking; wS1, whisker primary somatosensory cortex; wS2, whisker secondary somatosensory cortex. (TIF) [file pbio.3001667.s008.tif]

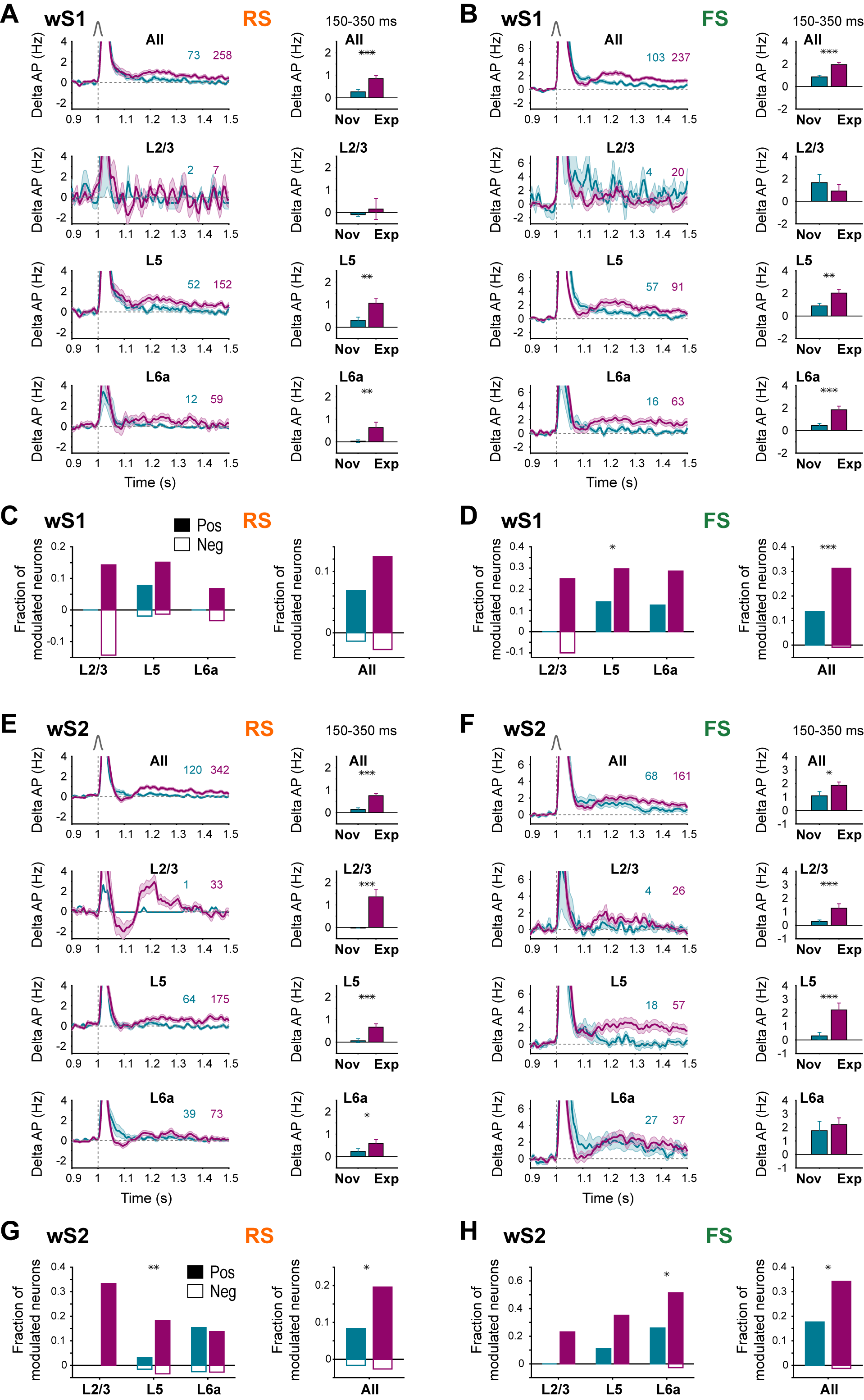

Supplement: S9 Fig — (A) Increase of late whisker response in wS1 RS neurons across learning. Left: baseline-subtracted (200 ms prior to whisker onset) population firing rate (mean ± SEM) for all neurons and different cortical layers (L2/3, L5, and L6a) separately overlaid for Novice and Expert mice. The number of neurons is indicated on the figure. Right: change in average spike rate quantified in 150- to 350-ms window after whisker onset relative to similar window size before whisker onset. ***: p < 0.001, **: p < 0.01, *: p < 0.05, ns: p > = 0.05, nonparametric permutation test, FDR-corrected for multiple comparison. (B) Increase of late whisker response in wS1 FS neurons across learning. Panels are similar to (A) but for wS1 FS neurons in Novice and Expert mice. (C) Fraction of wS1 RS neurons across different layers with significant positive (filled bars) or negative (empty bars) modulation late after whisker stimulus (150- to 350-ms window after whisker onset relative to similar window size before whisker onset). Positive or negative modulation of neurons was quantified using nonparametric permutation test (p < 0.005). ***: p < 0.001, **: p < 0.01, *: p < 0.05, ns: p > = 0.05, chi-squared proportion test. Fractions are reported for groups with more than 5 neurons. (D) Similar to (C) but for wS1 FS neurons. (E–H) Similar to (A–D) but for wS2. The underlying data for S9 Fig can be found in S6 Data. FDR, false discovery rate; FS, fast spiking; RS, regular spiking; wS1, whisker primary somatosensory cortex; wS2, whisker secondary somatosensory cortex. (TIF) [file pbio.3001667.s009.tif]

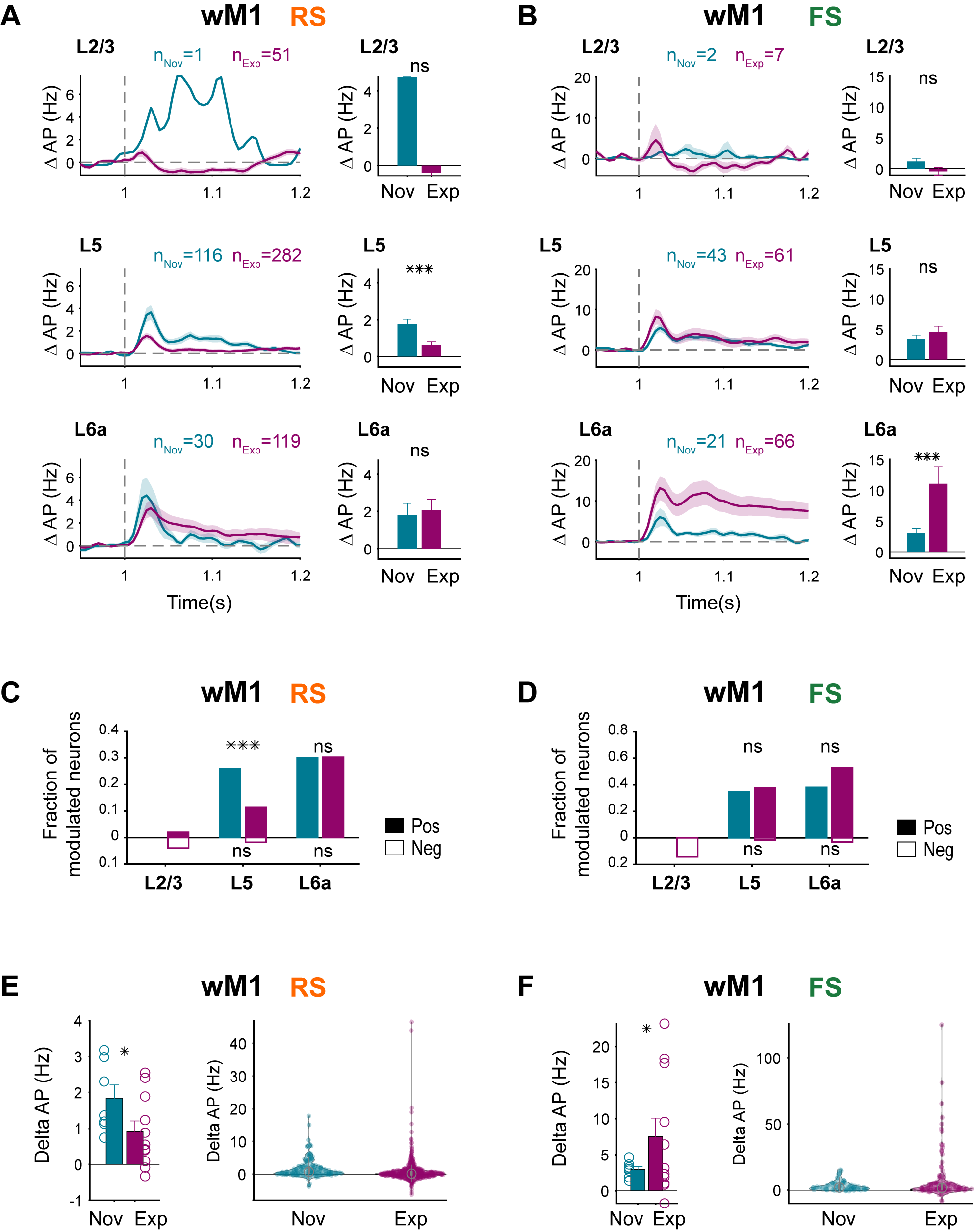

Supplement: S10 Fig — (A) Decrease of early whisker response in wM1 RS neurons across learning. Left: baseline-subtracted (50 ms prior to whisker onset) population firing rate (mean ± SEM) for different cortical layers (L2/3, L5, and L6a) overlaid for Novice (147 neurons in 7 mice) and Expert (452 neurons in 11 mice) mice. The number of neurons for each layer is indicated on the figure. Right: change in average spike rate quantified in 10- to 90-ms window after whisker onset relative to similar window size before whisker onset. ***: p < 0.001, ns: p > = 0.05, nonparametric permutation test, FDR-corrected for multiple comparison. (B) Increase of whisker response in wM1 FS neurons across learning. Panels are similar to (A) but for wM1 FS neurons in Novice (66 neurons in 7 mice) and Expert (134 neurons in 11 mice) mice. (C) Fraction of wM1 RS neurons across different layers with significant positive (filled bars) or negative (empty bars) modulation early after whisker stimulus (10- to 90-ms window after whisker onset relative to similar window size before whisker onset). Positive or negative modulation of neurons was quantified using nonparametric permutation test (p < 0.005). ***: p < 0.001, ns: p > = 0.05, chi-squared proportion test. Fractions are reported for groups with more than 5 neurons. (D) Similar to (C) but wM1 FS neurons. (E) Mouse-by-mouse variability and distribution of whisker-evoked response in RS units in wM1 of Novice and Expert mice. (Left) Bar plots showing average firing rate across mice in 10- to 90-ms window (mean ± SEM, 7 Novice and 11 Expert mice) after whisker onset and statistical comparison using nonparametric permutation test (*: p < 0.05). Circles show individual mice. (Right) Violin plots showing the distribution of whisker-evoked response in 10- to 90-ms window for all neurons recorded in Novice (147 neurons in 7 mice) and Expert mice (452 neurons in 11 mice). (F) Same as (E) but for wM1 FS units in Novice (66 neurons in 7 mice) and Expert mice (134 neurons i [file pbio.3001667.s010.tif]

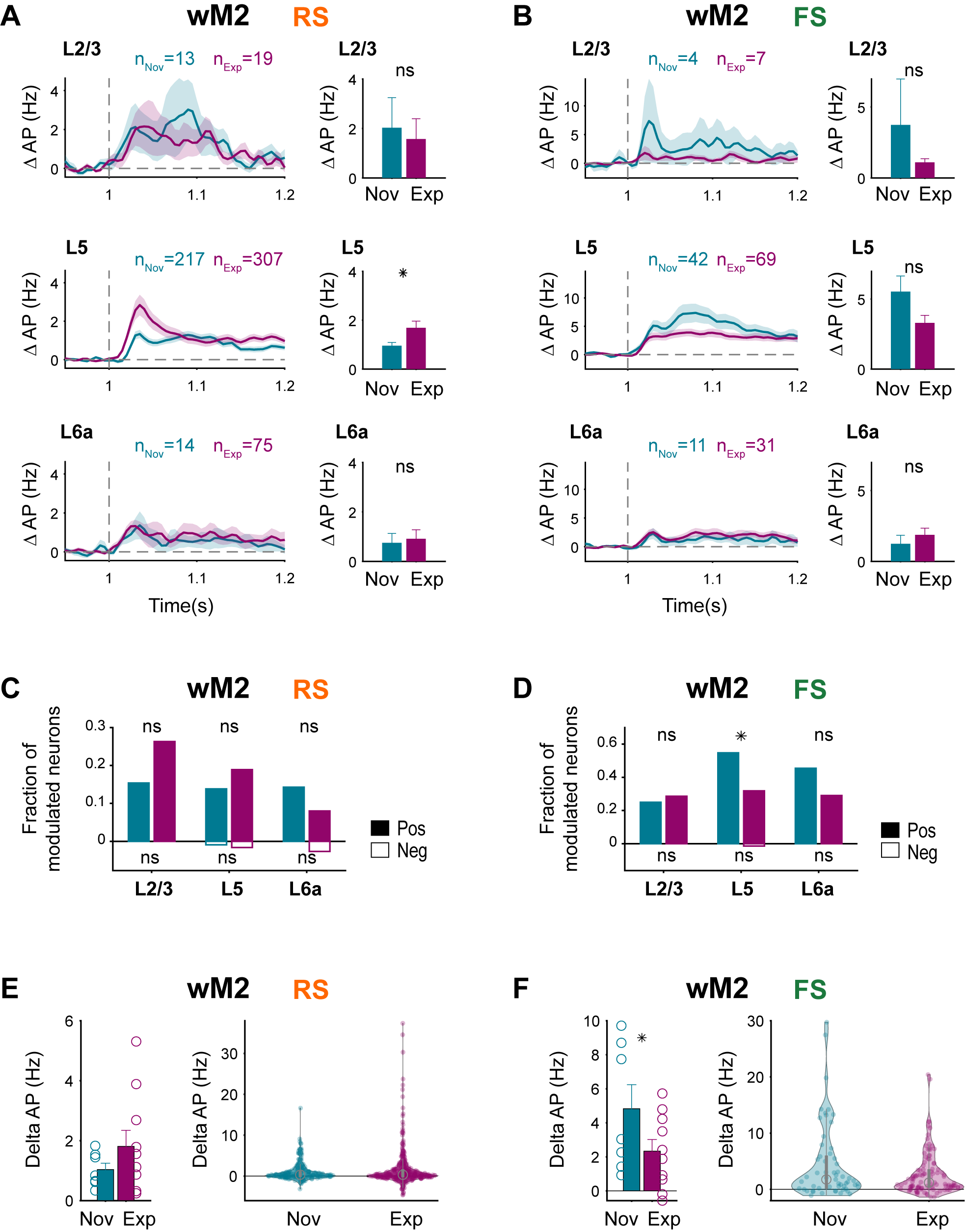

Supplement: S11 Fig — (A) Increase of early whisker response in wM2 RS neurons across learning. Left: baseline-subtracted (50 ms prior to whisker onset) population firing rate (mean ± SEM) for different cortical layers (L2/3, L5, and L6a) overlaid for Novice mice (244 neurons in 7 mice) and Expert mice (401 neurons in 11 mice). The number of neurons for each layer is indicated on the figure. Right: change in average spike rate quantified in 10- to 90-ms window after whisker onset relative to similar window size before whisker onset. *: p < 0.05, ns: p > = 0.05, nonparametric permutation test, FDR-corrected for multiple comparison. (B) Decrease of whisker response in in wM2 FS neurons across learning. Panels are similar to (A) but for wM2 FS neurons in Novice mice (57 neurons in 7 mice) and Expert mice (107 neurons in 10 mice). (C) Fraction of wM2 RS neurons across different layers with significant positive (filled bars) or negative (empty bars) modulation early after whisker stimulus (10- to 90-ms window after whisker onset relative to similar window size before whisker onset). Positive or negative modulation of neurons was quantified using nonparametric permutation test (p < 0.005). *: p < 0.05, ns: p > = 0.05, chi-squared proportion test. Fractions are reported for groups with more than 5 neurons. (D) Similar to (C) but wM2 FS neurons. (E) Mouse-by-mouse variability and distribution of whisker-evoked response in RS units in wM2 of Novice and Expert mice. (Left) Bar plot showing average firing rate across mice in 10- to 90-ms window (mean ± SEM, 7 Novice and 10 Expert mice) after whisker onset and statistical comparison using nonparametric permutation test (*: p < 0.05). Circles show individual mice. (Right) Violin plots showing the distribution of whisker-evoked response in 10- to 90-ms window for all neurons recorded in Novice (244 neurons in 7 mice) and Expert mice (401 neurons in 10 mice). (F) Same as (E) but for wM2 FS units in Novice (57 neurons in 7 mice) and Expert mice (107 neu [file pbio.3001667.s011.tif]

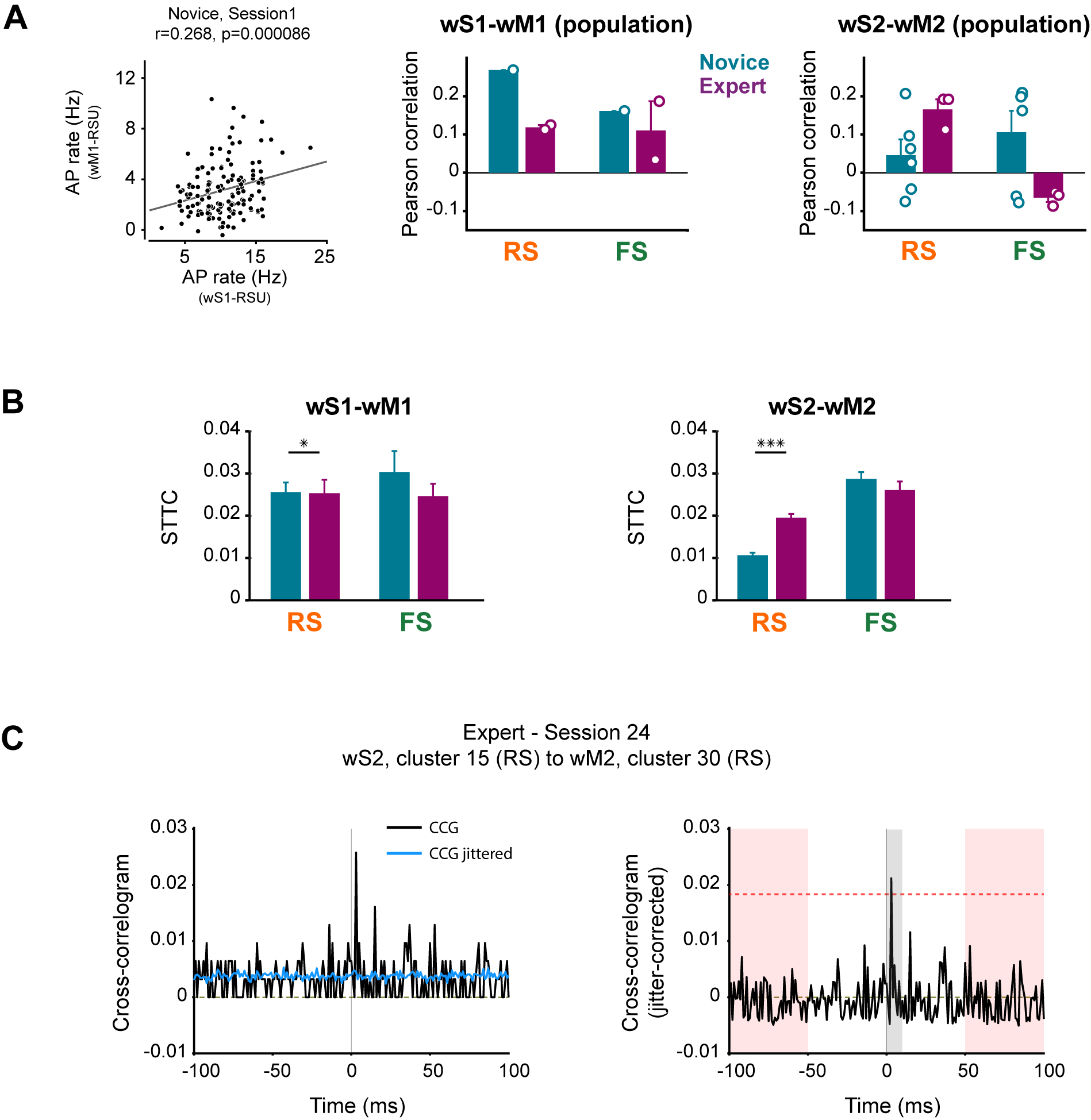

Supplement: S12 Fig — (A) Interareal correlation of population response in Novice and Expert mice. (Left) Scatter plot of trial-by-trial average population response between wS1-RS units and wM1-RS units for an example Novice session. Circles were jittered slightly for the purpose of visualization. Gray line: least-squares regression. (Middle) Pearson correlation of trial-by-trial average population response of wS1-RS units versus wM1-RS, and wS1-RS units versus wM1-FS units (1 Novice and 2 Expert mice). (Right) Pearson correlation of trial-by-trial population average response of wS2-RS units versus wM2-RS, and wS2-RS units versus wM2-FS units (6 Novice and 3 Expert mice). Circles show individual sessions. Error bars: SEM. (B) Pair-wise correlation between sensory and motor cortices in Novice and Expert mice using the STTC method. Left: Average pair-wise STTC correlation of wS1-RS units with wM1-RS (308 neuron pairs in 1 Novice mouse, and 398 neuron pairs in 2 Expert mice) and wS1-RS units with wM1-FS units (112 neuron pairs in 1 Novice mouse, and 139 neuron pairs in 2 Expert mice) separately. Right: Average pair-wise Pearson correlation of wS2-RS units with wM2-RS (3,482 neuron pairs in 6 Novice mouse, and 2,461 neuron pairs in 3 Expert mice) and wS2-RS units with wM2-FS units (821 neuron pairs in 6 Novice mouse, and 532 neuron pairs in 3 Expert mice). Error bars: SEM. Statistical comparison between Novice and Expert was performed using Wilcoxon rank-sum test (ns: p > = 0.05; *: p < 0.05; ***: p < 0.001). (C) Example cross-correlogram (CCG) from pair of neurons recorded simultaneously in wS2 and wM2 of an Expert mouse with a significant connection; same example pair as shown in Fig 6F, but with CCG from -100 to 100 ms time lags. Jitter correction method (left), and detection of significant functional connections (right). Significant connections were detected if any threshold crossing happened within 0- to 10-ms time lags (gray bar) of the jitter-corrected CCG. Threshold (red dotted line) [file pbio.3001667.s012.tif]

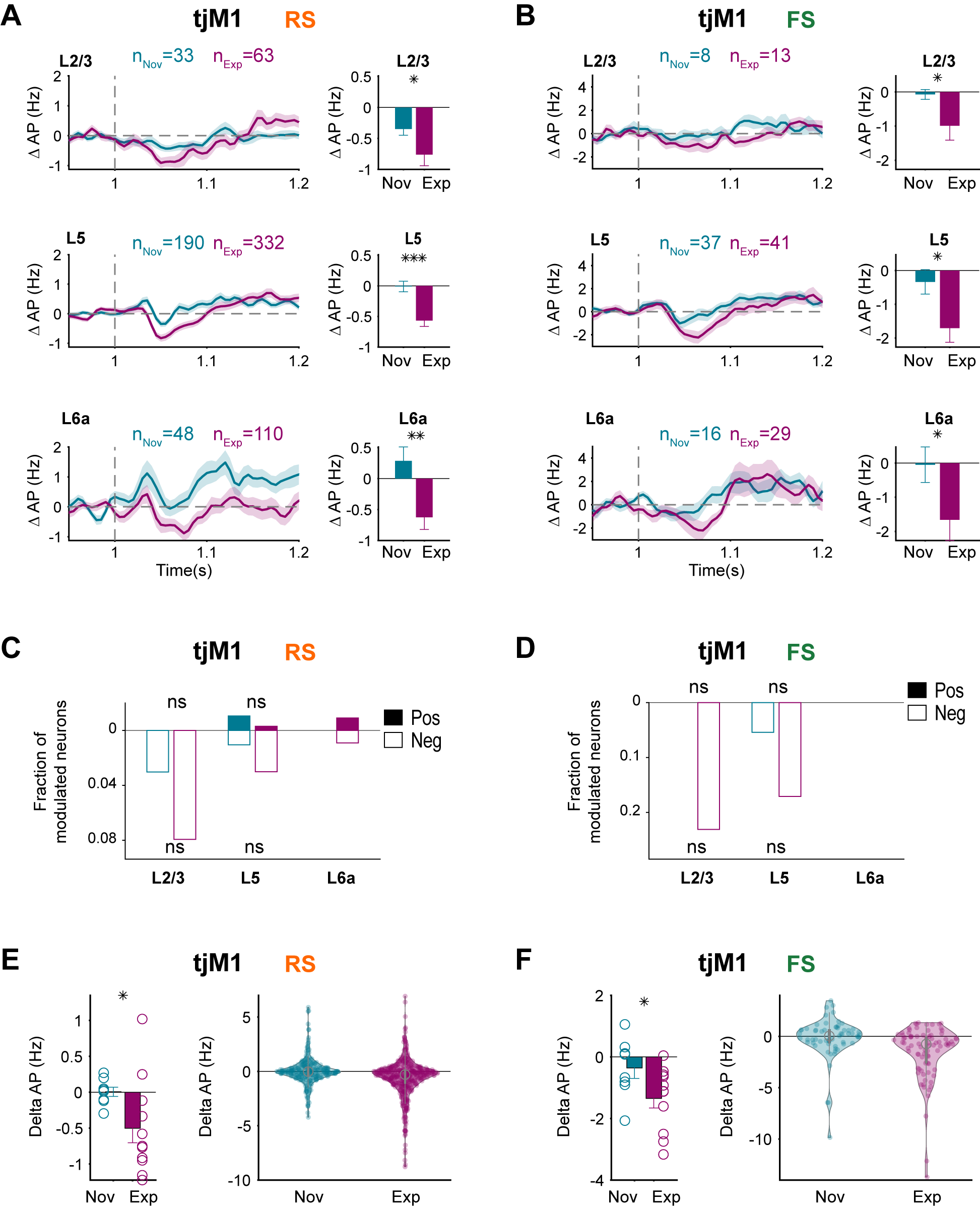

Supplement: S13 Fig — (A) Suppression of activity in tjM1 RS neurons across learning. Left: baseline-subtracted (50 ms prior to whisker onset) population firing rate (mean ± SEM) for different cortical layers (L2/3, L5, and L6a) overlaid for Novice mice (271 neurons in 8 mice) and Expert mice (505 neurons in 11 mice). The number of neurons for each layer is indicated on the figure. Right: change in average spike rate quantified in 40- to 90-ms window after whisker onset relative to similar window size before whisker onset. ***: p < 0.001, **: p < 0.01, *: p < 0.05, nonparametric permutation test, FDR-corrected for multiple comparison. (B) Suppression of activity in tjM1 FS neurons across learning. Panels are similar to (A) but for tjM1 FS neurons in Novice mice (61 neurons in 8 mice) and Expert mice (83 neurons in 11 mice). (C) Fraction of tjM1 RS neurons across different layers with significant positive (filled bars) or negative (empty bars) modulation early after whisker stimulus (40- to 90-ms window after whisker onset relative to similar window size before whisker onset). Positive or negative modulation of neurons was quantified using nonparametric permutation test (p < 0.005). ns: p > = 0.05, chi-squared proportion test. Fractions are reported for groups with more than 5 neurons. (D) Similar to (C) but tjM1 FS neurons. (E) Mouse-by-mouse variability and distribution of whisker-evoked response in RS units in tjM1 of Novice and Expert mice. (Left) Bar plots showing average firing rate across mice in 40- to 90-ms window (mean ± SEM, 8 Novice and 11 Expert mice) after whisker onset and statistical comparison using nonparametric permutation test (*: p < 0.05). Circles show individual mice. (Right) Violin plots showing the distribution of whisker-evoked response in 40- to 90-ms window for all neurons recorded in Novice (271 neurons in 8 mice) and Expert mice (505 neurons in 11 mice). (F) Same as (E) but for tjM1 FS units in Novice (61 neurons in 7 mice) and Expert mice (83 neurons in 11 m [file pbio.3001667.s013.tif]

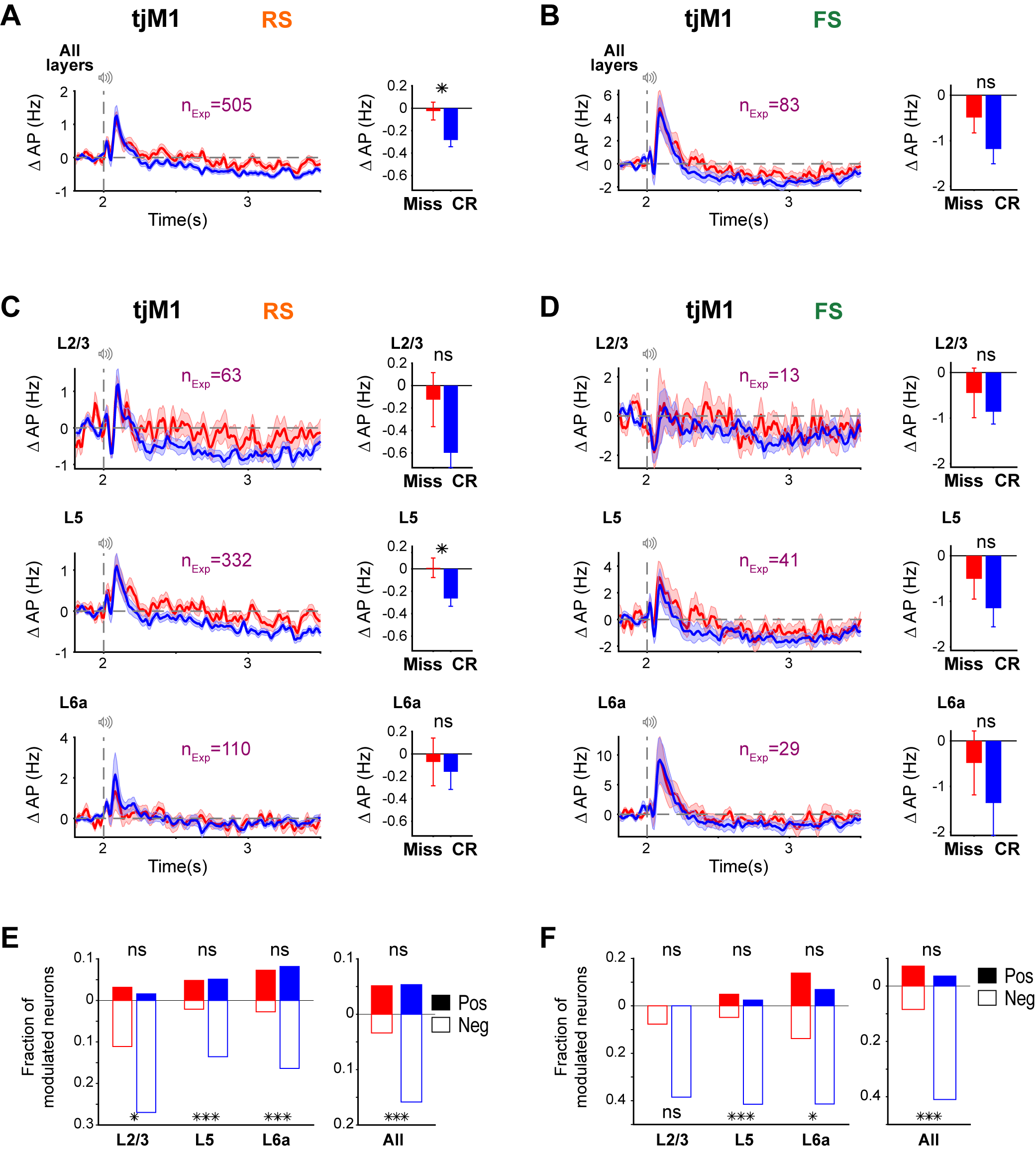

Supplement: S14 Fig — (A) Stronger suppression of tjM1 RS neurons in correct rejection versus miss trials. Left: baseline-subtracted (200 ms prior to auditory onset) population firing rate (mean ± SEM) overlaid for correct rejection (blue) and miss trials (red) in Expert mice (505 neurons in 11 mice). Right: change in average spike rate quantified in 200- to 1,000-ms window after auditory onset relative to a 200-ms window prior to auditory onset. *: p < 0.05, ns: p > = 0.05, nonparametric permutation test. (B) Similar to (A) but for FS neurons (83 neurons in 11 mice). (C) Similar to (A) but separately for RS neurons of different cortical layers. The number of neurons for each layer is indicated on the figure. *: p < 0.05, ns: p > = 0.05, nonparametric permutation test, FDR-corrected for multiple comparison. (D) Similar to (C) but for FS neurons. (E) Fraction of tjM1 RS neurons across different layers with significant positive (filled bars) or negative (empty bars) modulation in correct rejection and miss trials, quantified during response window (200- to 1,000-ms window after auditory onset relative to 200-ms window before auditory onset). Positive or negative modulation of neurons was quantified using nonparametric permutation test (p < 0.005). ***: p < 0.001, *: p < 0.05, ns: p > = 0.05, chi-squared proportion test. (F) Similar to (E) but tjM1 FS neurons. The underlying data for S14 Fig can be found in S9 Data. FDR, false discovery rate; FS, fast spiking; RS, regular spiking; tjM1, tongue-jaw primary motor cortex. (TIF) [file pbio.3001667.s014.tif]

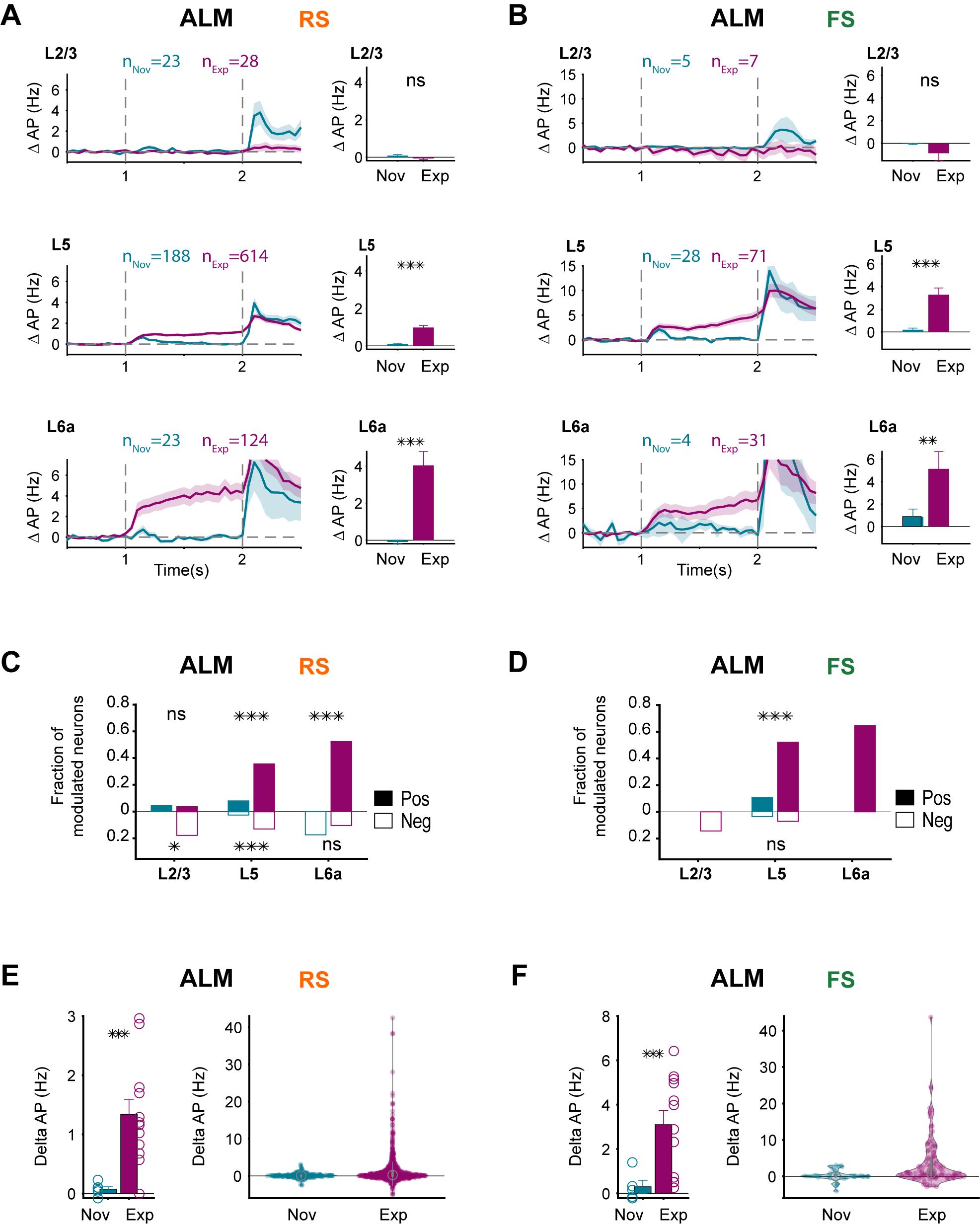

Supplement: S15 Fig — (A) Delay activity in ALM RS neurons upon learning. Left: baseline-subtracted (1 second prior to whisker onset) population firing rate (mean ± SEM) for different cortical layers (L2/3, L5, and L6a) overlaid for Novice mice (234 neurons in 6 mice) and Expert mice (766 neurons in 12 mice). The number of neurons for each layer is indicated on the figure. Right: change in average spike rate quantified in 200- to 1,000-ms window after whisker onset relative to similar window size before whisker onset. ***: p < 0.001, **: p < 0.01, ns: p > = 0.05, nonparametric permutation test, FDR-corrected for multiple comparison. (B) Delay activity in ALM FS neurons upon learning. Panels are similar to (A) but for ALM FS neurons in Novice mice (37 neurons in 5 mice) and Expert mice (109 neurons in 12 mice). (C) Fraction of ALM RS neurons across different layers with significant positive (filled bars) or negative (empty bars) modulation during delay period (200- to 1,000-ms window after whisker onset relative to similar window size before whisker onset). Positive or negative modulation of neurons was quantified using nonparametric permutation test (p < 0.005). ***: p < 0.001, *: p < 0.05, ns: p > = 0.05, chi-squared proportion test. Fractions are reported for groups with more than 5 neurons. (D) Similar to (C) but for ALM FS neurons. (E) Mouse-by-mouse variability and distribution of delay activity of RS units in ALM of Novice and Expert mice. (Left) Bar plots showing average firing rate across mice in 200- to 1,000-ms window (mean ± SEM, 6 Novice and 12 Expert mice) after whisker onset and statistical comparison using nonparametric permutation test (*: p < 0.05). Circles show individual mice. (Right) Violin plots showing the distribution of delay activity in 200- to 1,000-ms window for all neurons recorded in Novice (234 neurons in 6 mice) and Expert mice (766 neurons in 12 mice). (F) Same as (E) but for ALM FS units in Novice (37 neurons in 5 mice) and Expert mice (109 neurons in 12 [file pbio.3001667.s015.tif]

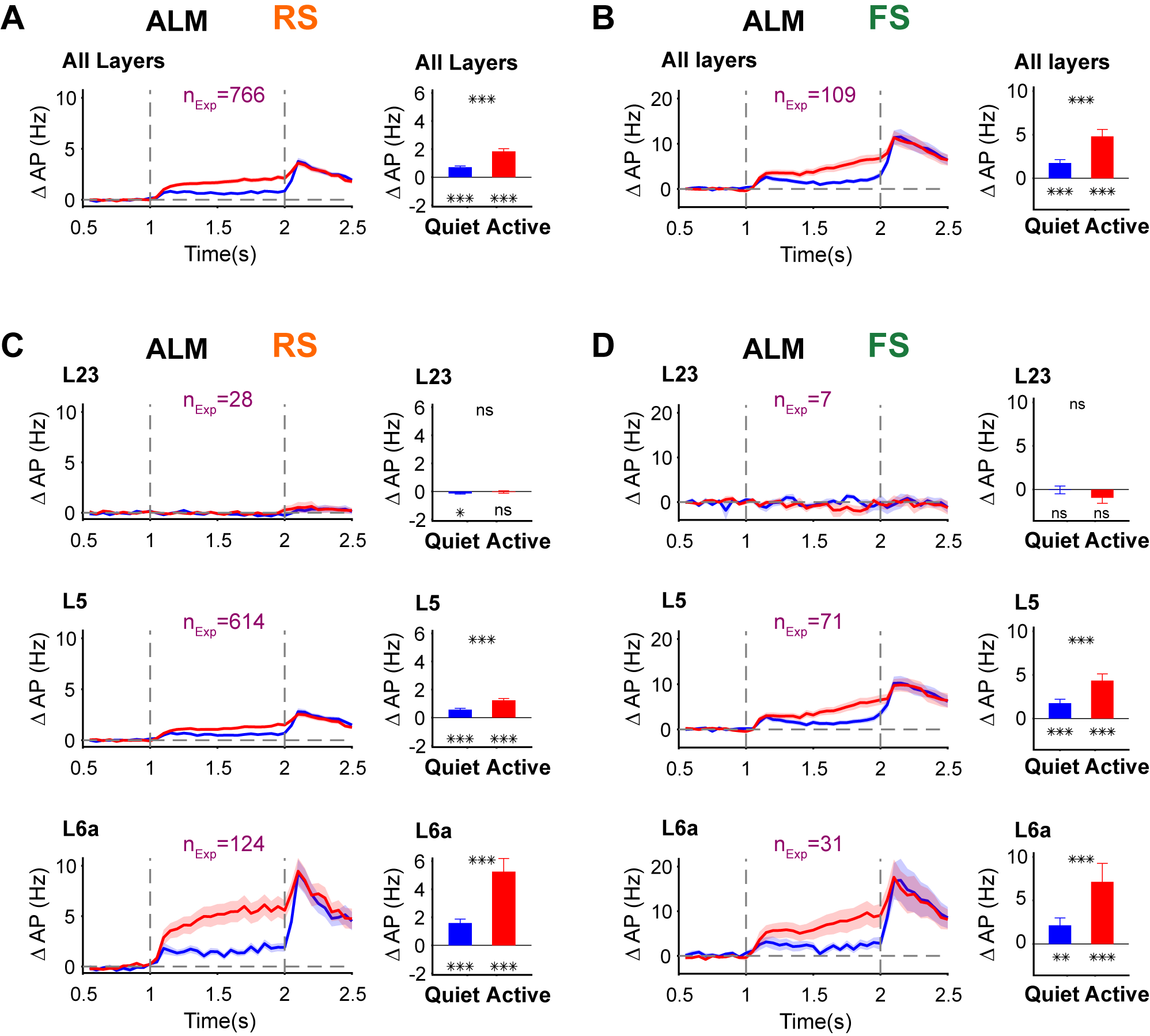

Supplement: S16 Fig — (A) Larger delay period activity of ALM RS neurons in Active versus Quiet hit trials. Left: baseline-subtracted (1 second prior to whisker onset) population firing rate (mean ± SEM) overlaid for Quiet (blue) and Active (red) hit trials in Expert mice (766 RS units in 12 mice). Right: change in average spike rate quantified in 200- to 1,000-ms window after whisker onset relative to a 1-second window prior to whisker onset. ***: p < 0.001, nonparametric permutation test. Asterisks below the bars represent the p-value for comparing delay activity in each trial type compared to baseline, while asterisks above the bars represent the p-value of the comparison between delay activity in Quiet and Active hit trials. (B) Similar to (A) but for FS neurons (109 FS units in 12 mice). (C) Similar to (A) but separately for RS neurons of different cortical layers. The number of neurons for each layer is indicated on the figure. ***: p < 0.001, ns: p > = 0.05, nonparametric permutation test, FDR-corrected for multiple comparison. (D) Similar to (C) but for FS neurons. The underlying data for S16 Fig can be found in S9 Data. ALM, anterior lateral motor cortex; FDR, false discovery rate; FS, fast spiking; RS, regular spiking. (TIF) [file pbio.3001667.s016.tif]
