# Supplementary material for: Learning-related congruent and incongruent changes of excitation and inhibition in distinct cortical areas
Source: PLoS Biol. 2022 May 31;20(5):e3001667. doi: 10.1371/journal.pbio.3001667 (PMC9187120; doi:10.1371/journal.pbio.3001667)
Supplement: Attachment — Submitted filename: Esmaeili_Exc_Inh_ReviewerReply_20220318.pdf [file pbio.3001667.s027.pdf]

Reviewer #1

Training rodents to report sensory percepts by licking a port for reward is a very commonly used experimental paradigm for investigating sensorimotor transformations, but knowledge about the differential roles played by excitatory and inhibitory neurons in such tasks is surprisingly lacking. The authors attempted to address this gap by examining the activity of putative excitatory (RS) and inhibitory (FS) neurons separately in a whisker-based delayed response task. They found that FS cells are strongly activated in all the cortical regions studied. In most cortical areas, the process of learning the task led to changes in FS cell activity that were similar to changes in RS cell activity. Interestingly, however, in two regions, wM1 and wM2, FS cell and RS cell activity changed in opposite directions due to learning. This finding also corroborates some observations from their previous paper investigating the same task.

Overall, the paper is of good quality and describes significant results that help fill important gaps in knowledge, and that are of general interest in systems neuroscience. Most of the claims made in the manuscript are backed up by the evidence presented, and the statistical analyses are satisfactory. My main complaints are that the authors could have taken some of the interesting findings a lot further.

Major points
1. The learning-related changes in activity of RS and FS cells in opposite directions in wM1 and wM2 were probably the most interesting and novel observations in the paper, but the authors did not pursue this avenue of inquiry fully. For example, what are the circuit-level mechanisms that bring these changes about? To what extent are they dependent on plasticity at synapses onto FS and RS neurons? Are the changes in the excitation/inhibition ratio at all important for the animal to perform the task? Whole cell recordings from identified cell types could help address some of these questions.

Several of the earlier figures summarize time-courses of FS and RS cell activity, but these findings don't seem to be particularly novel or unexpected. As the authors themselves acknowledge, FS cell activity patterns generally mirror RS cell activity, but with shorter latencies and larger amplitudes. While these are valuable results that should be reported, it is perhaps not surprising that this should be the case given that FS cells are well-known to form powerful feed-forward inhibitory connections within and between cortical regions. Instead, devoting more space to fully exploring the circuit-level processes underlying the changes in the excitation/inhibition balance in wM1 and wM2 would go a long way towards improving the manuscript.

We agree with the reviewer that the opposite changes of RS and FS neuronal activity comparing wM1 and wM2 is the most striking finding of this study. However, we think it is also important to report the apparent stability of the early sensory response of RS and FS neurons in wS1 and wS2, which provides an important contrasting result. We also think the congruent changes in RS and FS activity found in tjM1 and ALM across learning are also highly relevant for comparison. Indeed, we think it is the diversity of effects across learning observed in RS and FS neurons in different cortical areas, which is the strength of this study.

We also agree with the reviewer, that it will be important to investigate the underlying mechanisms. Whereas, we think that carrying out whole-cell recordings from identified cell-types is beyond the scope of the current paper, we have now carried out a series of analyses aiming to better understand the potential role of signaling from wS1->wM1 and wS2->wM2. In a subset of our recordings, we

simultaneously recorded from wS1 and wM1, and in another subset of experiments, we simultaneously recorded from wS2 and wM2. For these paired recordings, we carried out new analyses to study correlated trial-by-trial variations and spike-by-spike interactions:

i) In Figure 6E, we now present trial-by-trial covariations in whisker-deflection evoked action potential firing in the wS1->wM1 and wS2->wM2 pathways comparing between Novice and Expert mice. We found that trial-by-trial covariation of wS1->wM1 activity is not significantly changed across learning. However, the Pearson correlation of evoked activity of wS2-RS units and wM2-RS units is increased in Expert mice. This would be consistent with synaptic strengthening of wS2->wM2 pathway, which might contribute to the increased responses in wM2-RS units. Interestingly, the Pearson correlation of evoked activity of wS2-RS units and wM2-FS units is decreased in Expert mice, which could contribute to the reduced responses of wM2-FS units across learning. These single-trial correlation analyses are consistent with the hypothesis that synaptic plasticity changes could be occurring at direct monosynaptic projections from wS2 to wM2 and that different directions of synaptic plasticity onto RS and FS units could underlie part of the changes in responses in wM2, although other more complex explanations cannot be ruled out.

ii) In Figure 6F, we now present spike-by-spike correlations between neurons located in wS1 and wM1, and between neurons located in wS2 and wM2. We analysed all simultaneously recorded pairs of neurons focusing on RS units in wS1 and wS2, since excitatory neurons send direct long-range axonal projections to wM1 and wM2 respectively, as shown in Figure 5. We did not find a statistically-significant change across learning in the spike cross-correlograms for wS1->wM1 pairs. However, in paired recordings from wS2 and wM2, we did find an increase in the number of pairs with short-latency excitation of wM2-RS units in Expert mice compared to Novice mice. These analyses again point to increased coupling between excitatory neurons in wS2 and wM2, which would be consistent with long-term potentiation of the synapses between wS2-RS units and wM2-RS units, although again other more complex explanations cannot be ruled out.

We think these new analyses have strengthened the manuscript and provide the first steps towards more mechanistic investigations beginning to probe a potential role for synaptic plasticity between specific neuronal pathways in the learning of this whisker detection task.

2. Concerning Fig 4 E, the authors' claim of a differential effect on licking due to optogenetic inactivation of wS1 vs wS2 would be interesting, but this is not sufficiently backed up by the data. No controls for the optogenetic stimulation parameters were included - wS2 encompasses a smaller region of cortical surface than wS1, and so one might expect the same optical stimulation parameters to inhibit a much larger proportion of S2 neurons, thereby explaining the observed behavioral effect. Including some assays with different levels of optical stimulation intensities, and control experiments with stimulation of cortical areas unrelated to the task would help to make this part a lot more convincing.

The reviewer raises an important point. Neuronal activity related to a given task might be more localized or more distributed in different cortical areas, and the impact of optogenetic inactivation could be affected by this. This is a major point for almost any study using optogenetic inactivation methods, and it is a point that is extremely difficult to address.

As a first order approximation, we think the area of cortex involved in wS1 and wS2, might not be very different for this specific task. We only stimulate a single whisker (the C2 whisker) and the anatomically-defined C2 barrel column in wS1 is roughly 250 µm in diameter. Although in total the surface area of wS2 is smaller than wS1, the neurons have broader receptive fields, and so it is possible that C2-whisker responsive cells might be distributed over a similar surface area in wS1 and wS2. So we think it is likely that we inactivate the most important fraction of C2-responsive neurons in both wS1 and wS2 with our optogenetic inactivation, but we have not measured this, and so the reviewer's point remains to be fully addressed.

The same point could of course be made for any of the other cortical areas that we inactivated in this task (Esmaeili et al., 2021), and indeed could be applied to any other study using optogenetic inactivation. Although, we agree with the reviewer, that the results need to be interpreted cautiously, we nonetheless think that it is interesting to highlight the profound role of inactivating wS2, as well as wS1. In our previous paper (Esmaeili et al., 2021) we indeed inactivated other cortical regions and at different time points to reveal the spatiotemporal specificity of our manipulations.

In the text on P. 8, we now write: "*Optogenetic inactivation by applying blue light in VGAT-ChR2 mice to either wS1 and wS2 during the delivery of the whisker stimulus induced a significant decrease in hit rate [22]. Here, we reanalyzed this inactivation data in a direct comparison across these two areas, and found a significantly stronger deficit induced by inactivation of wS2 compared to wS1 (wS1: Δhit = -0.30 ± 0.13; wS2: Δhit = -0.49 ± 0.12; Wilcoxon signed-rank test, p = 0.0039; 9 mice) (Fig 4E). However, potential differences in the spatial extent of the whisker deflection-evoked responses and the efficacy of optogenetic inactivation in wS1 versus wS2 make it difficult to conclude the relative importance of sensory processing in these two areas. Nonetheless, the data suggest that neuronal activity in both wS1 and wS2 is involved in execution of this whisker detection task.*"

Minor points
1. The second paragraph of the Introduction seems to be somewhat broad. It would be helpful to add some more details about known roles of FS cells in cortex-dependent learning in order to help contextualize the results in the literature.

We have now added a brief discussion on FS cells in relation to learning. On P. 4 of the manuscript we now write: "*Previous whisker-related studies have reported experience-dependent plasticity of both excitatory and inhibitory synaptic transmission, with prominent changes reported in PV GABAergic neurons, for example following whisker deprivation [43,44]. However, it remains unknown how reward-based learning in whisker-dependent tasks might affect the activity of PV neurons, although previous work has revealed prominent changes in PV neuronal activity in mouse motor cortex during learning of a lever-press task [45] and in visual cortex during learning of a visual discrimination task [46].*"

2. It is not clear from the text whether Novice and Expert mice had overlapping sets of animals.

On P. 5 of the text in the revised manuscript, we now explicitly write: "*We recorded from two separate groups of mice referred to as "Novice" and "Expert" hereafter, …*"

3. Figure 4 C-D have missing x-axis labels.

We thank the reviewer for pointing this out. We have now corrected this in the revised version.

Reviewer #2

A major goal in neuroscience is to identify the neuronal circuits through which sensation is transformed into action. Esmaeili et al. has previously characterized how neurons along the sensori-motor hierarchy respond to stimuli as mice learn to report detection of whisker input. The current study reanalyzes this previous dataset to identify fast-spiking (FS; putative interneurons) and regular spiking (RS; putative pyramidal) cells and compare their strength and timing of recruitment across learning. The authors validate their classification of FS and RS cells using optotagging and comparisons of firing rates, and also retain appropriate caution in the assignment of cell types. Interestingly, they find that RS and FS cells change largely in parallel in sensory areas (wS1 and wS2) and in regions associated with motor output and licking (ALM and tjM1), but have opposite effects in whisker related motor areas (wM1 and wM2). Namely, RS neurons increase their activity relative to FS in wM2, but decrease their activity relative to FS neurons in wM1. This area- and cell-type specific plasticity is an exciting and novel example of how learning impacts cortical computations and will be of interest to a range of investigators studying circuits and behavior across systems and species. Overall, the study is well written and clearly presented (an accomplishment for a study with so many conditions) and I only have a few suggestions to improve robustness of the study.

1. The most important claim for the paper is the ability to use spike waveforms to identify putative inhibitory interneurons.

a. The optotagging approach is an important control to understand the potential incidence of false positives and false negatives. Thus, it would be preferable to include these data showing the relationship between spike width and ChR2 response in the main figures rather than the supplement. However, the methods provide limited information for how this data was collected and analyzed. Stimulus conditions are provided for the optrode recordings (although these are confusingly discussed in the context of wS1 inactivation) but not the LED-mediated activation, and no information is given for the analysis window that was used to measure firing rates. At least in the case of the optrode recordings, the stimulus window is long (600 ms) and therefore it might not be ideal to measure firing during the whole window as a proxy for ChR2 expression due to network effects (see for example Sanzeni et al., eLife, 2020).

We agree with the reviewer that opto-tagging is a potentially-powerful approach to study cortical circuitry in detail, and agree that the data need to be carefully interpreted. We have therefore followed the reviewer's suggestion and we have now included more of the VGAT-ChR2 opto-tagging analysis in a main figure (new panels in Figure 1J-O). In addition to expanding the previous analysis which quantified the light effect over a long window (400 ms) (Figure 1J-L), we have now also added a new analysis based on fast light-driven action potential firing focusing on the first 10-ms window after light onset (Figure 1M-O, Figure S1). As this analysis only considers the very early window after light stimulation onset, it should be largely devoid of network effects. In both analyses, we find that the vast majority of optically excited neurons have narrow action potential waveforms (FS units), whereas the vast majority of optically inhibited neurons have long-duration action potential waveforms (RS units). These further analyses thus support the notion that FS units are mainly inhibitory neurons.

We also carried out further new experiments to enable these analyses. In total we now report data from 5 sessions of 4 mice in the revised version. Importantly, we also ensured identical experimental conditions for the opto-tagging analyses, focusing exclusively on sessions with recordings using the standard silicon probe (of the type

used in all the non-opto-tagging experiments) where the light was delivered using an optical fiber (400 µm; NA = 0.39) coupled to a 470-nm high power LED.

On P. 5-6 of the revised manuscript, we now write: "*In a new set of recordings, we measured the impact of stimulating genetically-defined GABAergic neurons in mice expressing channelrhodopsin-2 (ChR2) under the control of the vesicular GABA transporter (VGAT) [46]. Blue light modulated the firing rate of RS and FS neurons in the opposite directions both at the level of population (Fig 1J) and at the level of individual neurons (Fig 1K). Overall, it increased the firing rate of FS units (blue light off: 4.3 ± 4.9 Hz; blue light on: 33.9 ± 32.7 Hz; 51 units recorded in 5 mice; non-parametric permutation test, $p < 10^{-4}$), whereas it decreased the spike rate of RS units (blue light off: 3.2 ± 3.4 Hz; blue light on: 1.3 ± 7.2 Hz; 130 units recorded in 5 mice; non-parametric permutation test, $p = 9x10^{-4}$) (Fig 1L). As a second approach, and to avoid network effects of light stimulation (Sanzeni et al., 2020), we focused only on the first 10-ms window after the onset of light stimulation and identified the opto-tagged neurons based on their fidelity of responses, response onset latency and jitter (Fig 1M-O and S4). A larger fraction of neurons was opto-tagged among FS neurons compared to RS neurons. These data are therefore consistent with the hypothesis that the majority of FS units are likely to be inhibitory neurons, whereas the majority of RS units are likely to be excitatory neurons.*"

The experimental conditions have now been clearly described in the methods section on P. 16, where we now write: "*Optogenetic tagging of GABAergic neurons. To evaluate to what extent the categorization of units as RS or FS based on spike width is useful for assessing the activity of excitatory versus inhibitory neurons, we performed simultaneous electrophysiological recordings and blue light stimulations in wS1 in 6 sessions from 5 VGAT-ChR2 mice, which express ChR2 in all GABAergic neuron types. A craniotomy was made over the C2 barrel column, identified by optical intrinsic imaging. In the first session for each mouse, a silicon probe (A1x32-Poly2-10mm-50 s-177-A32, NeuroNexus, MI, USA) was slowly lowered to a depth of ~1000 µm in the C2 barrel column, and an optic fiber (400 µm; NA = 0.39, Thorlabs) coupled to a 470 nm high power LED (M470F3, Thorlabs, USA) was positioned close to the brain surface and the probe. A 100 Hz train of blue light pulses (50% duty cycle, mean power 1-2 mW) with the duration of 600 ms was applied. Light pulse train was followed by an additional 100 ms ramping down to prevent rebound excitation. In total, 51 FS and 130 RS units were identified in 6 sessions from 5 mice.*"

b. As shown in Figure S2, the relative fraction of FS to RS cells varies substantially across areas. It is surprising that more than half of the cells in wS1 are characterized as FS (though notably this is only true for the behavioral dataset; the optotagging dataset is much more biased towards RS cells). Some of this likely reflects true differences in the density of these cell types across areas, but also likely reveals some sampling biases. The authors should provide some discussion for how such a bias might impact their results.

We agree with the reviewer that this is surprising, although apparently robust in our data set, because it was observed independently in both Novice and Expert mice. We agree with the reviewer that there are likely to be various factors that contribute to the apparently differential distribution of RS and FS units comparing sensory and frontal cortex:

i) If we consider FS units to be PV neurons, then one part of the explanation for the spatial differences in the prevalence of FS neurons in our recordings, might be that

the distribution of PV neurons across the cortical mantle is apparently not uniform. In the most complete characterization of the distribution of PV neurons in the mouse neocortex, Kim et al. (*Cell* 2017) found that:

MOp had 4060 $\pm$ 379 (Male) and 3880 $\pm$ 285 (Female) PV neurons / mm^3
MOs had 3763 $\pm$ 339 (Male) and 3598 $\pm$ 261 (Female) PV neurons / mm^3
SSp had 5617 $\pm$ 416 (Male) and 5418 $\pm$ 493 (Female) PV neurons / mm^3
SSs had 4837 $\pm$ 291 (Male) and 4659 $\pm$ 409 (Female) PV neurons / mm^3

These data suggest the sensory cortex might have ~30% more PV neurons than frontal cortex, which might thus contribute to the larger number of FS units identified in wS1/2 compared to more frontal areas in our recordings.

ii) The extracellular recording technique and identification of single units is biased to detect neurons that fire many action potentials. Sensory cortex is often characterized as having low firing rates, or sparse activity, perhaps enabled by strong local inhibition. The low firing rates of excitatory neurons in sensory cortex might be another important factor giving the impression of different numbers fewer RS units in sensory areas. Future improvements of the extracellular recording approach allowing reliable detection of neurons with lower firing rates might partially correct for such bias.

We now briefly discuss these points on P. 5, where we write: “Unexpectedly, we found a larger fraction of FS units in sensory areas compared to frontal areas (S2 Fig), which could in part reflect differential distribution of PV neurons [50] and in part might indicate the known sampling bias of extracellular recordings limited to high-firing neurons, whereas sensory cortex typically has rather sparse activity.”

c. Given that the authors have simultaneous recordings from both FS and RS cells, it would be worthwhile to test whether these identities could be corroborated by functional measures, for instance testing connectivity using cross-correlations. Clearly, this would not be a high-yield approach, but could provide some internal validation of their behavior dataset.

We performed analyses based on cross-correlations to assess potential intra-areal functional connectivity between RS and FS cells, similar to the analysis described later for assessing inter-areal functional connectivity (see response to Reviewer #3). While, this method was efficient in identifying excitatory connections, it failed in detecting inhibitory ones, perhaps because of the limitations of this analysis and our dataset (e.g. sparseness of the connections, relatively short recording durations compared to those used in in-vitro or anesthetized experiments for such analysis, longer time-scale dynamics of the cross-correlograms which tend to shift the cross-correlogram up). Therefore, our analyses were not conclusive and we have not included these results.

2. The authors provide some interpretation of the increase in relative excitation in wM2 as increasing the drive to downstream motor areas such as ALM, but there is little discussion for what the role of the decrease in excitation of wM1 might mean for the circuit or behavior.

The reviewer raises an interesting question, to which we do not have a good answer. One might speculate that wM1 is more involved in the control of whisker movement, which is not required for task performance. Suppressing whisker movements, through reduced activity in wM1 in Expert mice compared to Novice mice, might help reduce sensory reafference signals evoked by self-generated whisker movements, which might in turn hinder the detection or short-term memory of the whisker stimulus. This

speculation is now briefly mentioned on P. 13, where we write "*We speculate that suppression of activity in wM1 could enhance whisker detection performance by reducing whisker movements [61], which otherwise could cause confounding sensory-reafference signals.*"

3. All of the data provided in the study are the averages of many neurons from multiple animals. The main figures should provide a better sense of the variability/robustness in the dataset: e.g. the distribution of firing rates across the population and/or comparisons of FS and RS cells on a mouse-by-mouse basis.

We now include the mouse-by-mouse data as well the distribution of firing rates across the population of recorded neurons in the manuscript in supplementary figures (Fig S10E-F, S11E-F, S13E-F, S15E-F).

4. It is not clear if the optogenetic inactivation is new data in this manuscript.

The optogenetic inactivation data presented in Figure 4 is part of a previously published dataset (Esmaeili et al., 2021) in which we assessed the impact of inactivation of several cortical regions (including wS1 and wS2) over different task epochs (Baseline, Whisker, Delay and Response windows). This data has been reanalyzed here, to directly compare the impact of inactivation of wS1 and wS2 during the early window after whisker presentation. We now explicitly write on P. 8: "*Optogenetic inactivation by applying blue light in VGAT-ChR2 mice to either wS1 and wS2 during the delivery of the whisker stimulus induced a significant decrease in hit rate [22]. Here, we reanalyzed this inactivation data in a direct comparison across these two areas, and found a significantly stronger deficit induced by inactivation of wS2 compared to wS1 (wS1: Δhit = -0.30 ± 0.13; wS2: Δhit = -0.49 ± 0.12; Wilcoxon signed-rank test, $p = 0.0039$; 9 mice) (Fig 4E). However, potential differences in the spatial extent of the whisker deflection-evoked responses and the efficacy of optogenetic inactivation in wS1 versus wS2 make it difficult to conclude the relative importance of sensory processing in these two areas. Nonetheless, the data suggest that neuronal activity in both wS1 and wS2 is involved in execution of this whisker detection task.*"

5. The x-axis of the time-courses are in trial time- it would be useful to have additional cues to remember what the relevant events are (e.g. whisker stim) in each figure.

We thank the reviewer for this suggestion and we have tried to add more information to the revised figures in order to help the reader.

Reviewer #3

The work of Esmaeili and colleagues reports the learning-related changes in whisker sensory, whisker motor and orofacial motor areas. This work builds on findings reported in a recent publication from Petersen lab. They further analyzed the data set of single unit recordings made simultaneously in six cortical regions by resolving changes in firing rates of specific neuronal subtypes - putative excitatory and inhibitory neurons. Main findings of the work are following. Little learning-related changes were observed in whisker primary and secondary somatosensory areas (wS1, wS2), although microcircuit-level changes could not be resolved in the study. In whisker primary and secondary motor areas (wM1 and wM2), excitatory and inhibitory neurons changed whisker-evoked responses in opposite directions. In wM1, inhibitory neurons increased firing rate whereas excitatory neurons decreased firing rate across learning. In wM2, inhibitory neurons decreased firing rate while excitatory neurons increased firing rate. In orofacial areas, excitatory and inhibitory neurons showed congruent changes because both subtypes either increased (in ALM) or decreased (in tjM1) the whisker evoked activity. This is an important analysis of data set acquired in a well-conducted study. The text is concise and generally clear, figures are of a good quality. However, I have several comments and suggestions.

Major points:
In its present form, the present study is focused on describing learning-related changes of excitation and inhibition in key cortical regions involved in the task but falls short of providing mechanistic insights into how changes in one area are related to those in another area. Figure 5 shows anatomical data suggesting that learning-related changes in wM2 are driven by wS2. If this is the case, activities in wS2 and wM2 would show enhanced coordination in expert mice. Establishing a causal relationship between wS2 and wM2 ultimately requires manipulation of wS2 while recording from wM2, which would be beyond the scope of the study. Nonetheless, one could use 'spike-triggered' approach as described in 2015 Neuron paper by Zandvakili and Adam Kohn where they showed V2 activity was preceded by increased coordination in V1 activity. Alternatively, one could infer a causal relationship using granger causality as described in the Neuron paper from Patrick Kanold's lab (Francis et al., 2018). Without providing additional information on functional changes in coordinated activity across cortical regions, I feel that the authors are not making full use of the excellent data set they collected and that Figure 5 is disjointed from the rest of the manuscript.

We thank the reviewer for this interesting suggestion. As described above in our reply to Major Point #1 of Reviewer #1, we have performed two new sets of analyses to explore learning-related changes in functional connectivity between wS2 and wM2 (Fig 6 and Fig S12):
i) First, taking advantage of trial-by-trial variability of evoked responses, we quantified the correlation between wS2 and wM2 neurons – both at the level of pair of individual neurons and average population response. Comparing Novice and Expert mice, the correlation of wS2-RS to wM2-RS units increased, whereas the correlation of wS2-RS to wM2-FS units decreased. These learning-related changes were not found between wS1 and wM1 neurons (Fig 6E and Fig S12A).
ii) Secondly, we used a cross-correlation approach, to assess the inter-areal functional connectivity. We found significantly larger fraction of connections from wS2-RS to wM2-RS of Expert mice (Figure 6F and Fig S12B).

On P. 10 of the manuscript we now write: "*To test how the coordination between sensory and motor cortices changed across learning, we quantified inter-areal interactions between wS1-wM1 and wS2-wM2 in a subset of sessions with*

simultaneous paired recordings (Fig 6E and 6F). Averaged over individual pairs of neurons, trial-by-trial correlation between wS2-RS units with wM2-RS units increased across learning (Novice: 876 neuron pairs recorded in 6 mice, Expert: 583 neuron pairs recorded in 3 mice; Wilcoxon rank-sum test, p = 0.039) while it decreased for wM2-FS units (Novice: 343 neuron pairs recorded in 6 mouse, Expert: 209 neuron pairs recorded in 3 mice; Wilcoxon rank-sum test, p = 2.9x10-4). Correlations between wS1-wM1 neurons did not change significantly. Trial-by-trial correlation in population response showed similar patterns of change across learning in both area pairs as those observed in pair-wise correlation changes (Fig S12A). To further evaluate functional connectivity changes, we identified the number of directional connections based on short-latency sharp peaks in the cross-correlograms between pairs of neurons from whisker sensory and whisker motor cortices (Fig 6F and S12B). The percentage of connections between wS2-RS units to wM2-RS units increased significantly across learning (Novice: 3 out of 1077 pairs in 6 mice, Expert: 17 out of 1066 pairs in 3 mice; proportion test, p = 0.0032).

All together, these data suggest that learning might increase the excitation to inhibition ratio of the sensory-evoked response in wM2, but decreases the ratio in wM1 in favor of inhibition. Increased activity of excitatory neurons in wM2 across learning could arise from the increase in functional connectivity between wS2 to wM2, and could in turn contribute to driving excitation in other frontal areas including ALM, which is known to be important for the motor planning of licking [20,22]."

Minor points:
1. RS and FS units in wS1: Supp. Fig. 3 shows no significant difference in AP rates of RS and FS units in wS1. This is a bit puzzling to me because FS units are known to show higher firing rate. Consistent with this, Supp. Fig. 4 and Fig. 1L show elevated firing rate of FS units before the onset of photostimulation. This does not impact the conclusion of the paper, but is worth clarifying.

The reviewer makes an interesting point. On average, across recorded areas, we did find that FS units fire at higher rates than RS units in the baseline period. However, the reviewer is correct to point out that we did not find significantly elevated baseline firing for FS units compared to RS units in wS1. The distribution of firing rates for both RS and FS units is very broad, so in part this may result from our limited data sample. Furthermore, low firing units are unlikely to be identified in extracellular recordings followed by spike sorting, because of the need for a large number of action potentials to be recorded. Extracellular recordings might thus give a biased representation of spiking, and specifically might not appropriately quantify the number of low firing RS units in wS1, which is known for sparse firing.

2. On page 7, paragraph 1, it is stated that "fast dynamic changes in the relative timing of the recruitment of FS and RS units across cortical regions". The statement implies that the changes occur across cortical regions, although they only occur in wM1 and wM2 (Figure 3). The statement could be toned down a bit.

We have followed the reviewer's suggestion and now write "*These latency differences reveal that task learning is accompanied by fast dynamic changes in the relative timing of the recruitment of FS and RS units across wM1 and wM2.*"

3. Figure 2: a plot showing first lick probability in novice and expert animals could be included and aligned to the firing rate traces.

We have followed reviewer's suggestion and have added lick histograms in novice and expert mice aligned to the firing rate traces in revised Fig 2.

4. Figure 4 focuses on fast whisker-evoked responses (50 ms after the stimulus onset). No significant changes were observed in the response of RS or FS units in wS1 and wS2 during this short time window. Petersen lab previously reported the presence of 'late' depolarization or excitation that emerges learning-dependent manner. These 'late' responses are clearly shown in average traces of FS and RS activities (Figure 2) but were not analyzed. Could the analysis of AP rate change (Fig. 4C) be extended to the 'late' component of the response for both wS1 and wS2?

We have followed the reviewer's recommendation and have added a new supplementary figure (Figure S9), which shows overlay of the firing rate traces in Novice and Expert of wS1 and wS2, as well as the quantifications in the "late" window (150-350 ms after whisker stimulus). Consistent with earlier reports in whisker detection tasks without a delay, here we also find an enhanced late secondary response which develops with learning.

5. Figure 5: do any of S2-->M2 projections arrive in ALM or M1? Suter and Shepherd 2015 showed that wS2 and wM1 are reciprocally connected, so I would expect some projections in wM1. Quantification of projection fields would be helpful to address these points.

The reviewer is correct to point out that the projections are less specific than they appeared in the original figure representation. In the previous version of the manuscript, we only showed a 95% contour of axon density, which appeared highly localized, with wS1 specifically targeting wM1 and wS2 specifically targeting wM2. In fact, as the reviewer points out, the axons actually innervate a much broader zone in frontal cortex, and to indicate the overlap in the parallel frontal projection pathways, we have now included a 75% axon density contour in the revised Figure 5H.

6. Supp. Fig. 13: there is a typo in the heading. "RS and FS neuronal activity in tjM1" --> "....in ALM"

We thank the reviewer for pointing this out, and we have corrected this in the revised manuscript.

Reviewer #4

In their paper 'Learning-related congruent and incongruent changes of excitation and inhibition in distinct cortical regions' Esmaeili et al. describes changes in fast spiking and regular spiking neurons during perceptual learning across a set of cortical areas in the mouse. This study ties in with a large body of work that the authors performed around similar tasks in mice. The experiments are performed to a high experimental standard, there appear to be no animal welfare issues. The key finding is that fast spiking and regular spiking neurons do not show the same learning related changes across cortical areas. I do think this is a potentially important result. Most of the other findings are minor. I think it would be great if the authors would provide a bit more background on their key finding, otherwise I think this study suitable for publication.

In the introduction, we have now expanded discussion on the plasticity of PV neurons reported in previous studies to provide more relevant background. On P. 3-4, we now write: "*Neocortex has regional specializations and a columnar organization divided into layers each containing many classes of neurons varying across diverse features [24–28]. At the most basic level, neocortical neurons can be classified as excitatory (releasing glutamate) or inhibitory (releasing GABA). Many neocortical excitatory neurons send long-range axons projecting to diverse brain regions, whereas most neocortical inhibitory neurons only have local axonal arborisations, thus contributing primarily to the regulation of local microcircuit activity. The balance between excitation and inhibition is likely to have a major impact on neocortical microcircuit computations and previous work has suggested important changes in this balance across development, brain states, sensorimotor processing and models of brain diseases [29–36]. Inhibitory GABAergic neurons can be further divided into many subclasses, with one of the most prominent being the parvalbumin-expressing (PV) neurons. PV cells provide potent inhibition onto excitatory cells by prominently innervating either the soma and proximal dendrites or the axonal initial segment, thus playing a critical role in controlling the discharge of excitatory neurons. At the millisecond timescale, the PV neurons appear specialized for high-speed synaptic computations with fast membrane time-constants and large fast synaptic conductances, receiving substantial excitatory input from many nearby excitatory neurons as well as long-range inputs [37–42]. Within a neocortical microcircuit, PV neurons are likely to play a critical role in controlling the balance between excitation and inhibition. PV cells typically fire at high rates and have short action potential (AP) durations that can easily be identified from extracellular recordings. In fact, neurons recorded from extracellular recordings are typically classified based of their AP duration, as regular spiking (RS) units, which have broad AP waveforms and correspond mostly to excitatory neurons; and fast spiking (FS) units, which have narrow AP waveforms and largely correspond to inhibitory PV neurons. Previous whisker-related studies have reported experience-dependent plasticity of both excitatory and inhibitory synaptic transmission, with prominent changes reported in PV GABAergic neurons, for example following whisker deprivation [43,44]. However, it remains unknown how reward-based learning in whisker-dependent tasks might affect the activity of PV neurons, although previous work has revealed prominent changes in PV neuronal activity in mouse motor cortex during learning of a lever-press task [45] and in visual cortex during learning of a visual discrimination task [46].*"

Major

Are the interneurons the same across the cortical regions investigated?

We think it is likely that similar classes of GABAergic neurons are present in different neocortical areas. From a developmental perspective, GABAergic neurons originate from ganglionic eminences and subsequently migrate laterally into the neocortex (Lim et al., Neuron 2018). Although there is surely some level of specialization of the GABAergic neurons in different cortical areas, they all originate from the same developmental process irrespective of where they finally end up integrating into the maturing neocortical microcircuits. From another perspective, it has been found through single cell RNA sequencing data, that the classes of GABAergic neurons found in frontal cortex are similar to those found in visual cortex, although for the excitatory neurons much more diversity has been reported (Tasic et al., Nature 2018).

It would be great if the authors could add info on the laminar distribution of their effects.

We thank the reviewer for his interest in layer-specific activity. We show laminar distributions for many of the results reported in main figures in the supplemental figures. Supplemental Figure 8 covers layer-specificity in wS1 and wS2 for fast sensory responses and Supplemental Figure 9 covers layer-specificity in wS1 and wS2 for the late secondary responses. Supplemental Figure 10 covers layer specificity in wM1 and Supplemental Figure 11 for wM2. Supplemental Figures 13 and 14 cover layer specificity in tjM1. Supplemental Figures 15 and 16 cover layer specificity in ALM.

What is the author's idea on the functional significance of their finding?

The long-term goal of our research is to define the synaptic and circuit mechanisms accounting for the execution and learning of simple whisker perceptual tasks. Our data suggest the involvement of neurons located in various distinct brain regions (Esmaeili et al., 2021), and obtaining mechanistic insight is challenging due to the large scale of the apparently contributing networks. We think the data in the current manuscript further support the hypothesis that important sensory information is transferred from wS2 to wM2 for the execution of this delayed whisker-detection task. Our data point to strengthened functional connectivity from wS2 to wM2 in Expert mice. In part this could result from processes such as long-term synaptic potentiation of glutamatergic input onto RS units in wM2, causing a direct increase in their responsivity. However, our data also reveal reduced recruitment of FS units in wM2, which could therefore contribute to gating the response of the excitatory neurons. Our current work, therefore begins to pinpoint interactions between specific neuronal classes as potential substrates for task learning, but further experiments are necessary to gain deeper insight into exactly which synapses undergo plasticity and contribute to the learning process.

Minor

Title: are we talking about cortical regions or cortical areas?

We are not sure of the precise difference between ‘*regions*’ and ‘*areas*’, but we think that ‘*areas*’ might refer to a more localized and specific parcellation of cortex. We therefore changed the title to refer to ‘areas’ rather than ‘regions’ because we investigate closely-neighbouring parts of cortex.

Figure 2: The annotation hit is slightly confusing; replace with 'hit trials' or 'hit trials only'?

Following reviewer’s suggestion, we have changed ‘Hit’ to ‘Hit trials’ in the revised version of Figure 2.
