# Supplementary material for: Learning-related congruent and incongruent changes of excitation and inhibition in distinct cortical areas
Source: PLoS Biol. 2022 May 31;20(5):e3001667. doi: 10.1371/journal.pbio.3001667 (PMC9187120; doi:10.1371/journal.pbio.3001667)
Supplement: Attachment — Submitted filename: Esmaeili_Exc_Inh_ReviewerReply_20220503.pdf [file pbio.3001667.s028.pdf]

EPFL | Switzerland
3rd May 2022

Reviewer #2 noted that the correlation "*measures are extremely sensitive to overall firing rates, which also change across learning, and the authors have not performed the necessary control analyses to ensure that this can not explain the observed effects*". We agree with the counter-points raised by Reviewer #3, relating to the absence of correlation changes in wS1->wM1-FS although firing rates also increased in this areal-pair. We examined our data base, and we did not have further areas simultaneously recorded together with wS2, so we cannot carry out the further 'control' analysis suggested by Reviewer #3. We did however follow the suggestion of the Academic Editor, who suggested using the method of spike time tiling coefficient (STTC), and this analysis is now included in Supplementary Figure S12B. The STTC analysis indicated a significant increase for wS2->wM2-RS units, consistent with our previous analyses. This further analysis has therefore strengthened the study and we thank the Reviewers and Academic Editor.

Reviewer #4 noted that Figure 1 could be split into two smaller figures, but we think this would disrupt some aspects of continuity, and we have therefore left the figure intact. We can of course split the figure if you nonetheless think this is best.

EPFL Neuroscience | Brain Mind Institute
Laboratory of Sensory Processing
School of Life Sciences

Prof. Carl Petersen
EPFL SV BMI LSENS
Station N° 19
CH - 1015 Lausanne
Switzerland

carl.petersen@epfl.ch
https://www.epfl.ch/schools/sv/bmi/
https://www.epfl.ch/labs/lsens/
